# Practice recommendations for the role of physiotherapy in the management of sleep disorders: the 2022 Brazilian Sleep Association Guidelines

**DOI:** 10.5935/1984-0063.20220083

**Published:** 2022

**Authors:** Cristina Frange, Aline Marques Franco, Evelyn Brasil, Raquel Pastrello Hirata, Juliana Arcanjo Lino, Daiana Moreira Mortari, Daisy Satomi Ykeda, Mario André Leocádio-Miguel, Carolina Vicaria Rodrigues D’Aurea, Luciana Oliveira e Silva, Susana Cristina Lerosa Telles, Sofia Fontanello Furlan, Bruno Búrigo Peruchi, Camila Ferreira Leite, Fabiana Tokie Yagihara, Leticia Dominguez Campos, Melissa Araújo Ulhôa, Moara Gomes da Rocha Cruz, Ricardo Beidacki, Ronaldo Batista Santos, Sandra Souza de Queiroz, Simone Barreto, Vivien Schmeling Piccin, Fernando Morgadinho Santos Coelho, Luciana Studart, Marcia Assis, Luciano F. Drager

**Affiliations:** 1 Departamento de Neurologia e Neurocirurgia, Escola Paulista de Medicina (EPM), Universidade Federal de São Paulo (UNIFESP) - São Paulo - SP - Brazil; 2 Associação Brasileira do Sono - São Paulo - SP - Brazil; 3 Departamento de Neurociências e Ciências do Comportamento, Faculdade de Medicina de Ribeirão Preto, Universidade de São Paulo (FMRP-USP) - Ribeirão Preto - SP - Brazil; 4 Departamento de Terapia Intensiva, Hospital Israelita Albert Einstein (HIAE) - São Paulo - SP - Brazil; 5 Laboratório de Pesquisa em Fisioterapia Pulmonar, Departamento de Fisioterapia, Universidade Estadual de Londrina (UEL) - Londrina - PR - Brazil; 6 Ciências Médicas, Universidade Federal do Ceará (UFC) - Fortaleza - CE - Brazil; 7 Universidade Federal do Rio Grande do Sul - Porto Alegre - RS - Brazil; 8 Curso de Fisioterapia, Universidade Estadual do Piauí (UESPI) - Teresina - PI - Brazil; 9 Departamento de Fisiologia e Comportamento, Universidade Federal do Rio Grande do Norte - Natal - RN - Brazil; 10 Departamento de Ortopedia, HIAE - São Paulo - SP - Brazil; 11 Departamento de Ciências da Saúde, Universidade Federal de Uberlândia (UFU) - Uberlândia - MG - Brazil; 12 Unidade de Hipertensão, Instituto do Coração (InCor), Faculdade de Medicina, Universidade de São Paulo (USP) - São Paulo - SP - Brazil; 13 Laboratório de Neurociência, Universidade do Estado de Santa Catarina (UNESC) - Criciúma - SC - Brazil; 14 Departamento de Fisioterapia, UFC; Programas de Mestrado em Fisioterapia e Funcionalidade, e Mestrado em Ciências Cardiovasculares, UFC - Fortaleza - CE - Brazil; 15 Setor de Neurofisiologia Clínica, Departamento de Neurologia e Neurocirurgia, EPM, UNIFESP - São Paulo - SP - Brazil; 16 Faculdade Inspirar - Bauru - SP - Brazil; 17 Instituto Metropolitano de Ensino Superior, Faculdade de Medicina do Vale do Aço (UNIVAÇO) - Ipatinga - MG - Brazil; 18 Faculdade Inspirar, Unidade Porto Alegre - Porto Alegre - RS - Brazil; 19 Departamento de Fisioterapia, Hospital Universitário, USP - São Paulo - Brazil; 20 Instituto do Sono, Associação Fundo Incentivo à Pesquisa - São Paulo - Brazil; 21 Laboratório do Sono, Divisão de Pneumologia do Instituto do Coração (InCor), FMUSP, USP - São Paulo - SP - Brazil; 22 Departamento de Psicobiologia, EPM, UNIFESP - São Paulo - SP - Brazil; 23 Universidade Federal de Pernambuco - Recife - PE - Brazil; 24 Clínica do Sono de Curitiba, Hospital São Lucas - Curitiba - PR - Brazil

**Keywords:** Sleep, Sleep Disorders, Physiotherapy, Rehabilitation, Sleep Health

## Abstract

This clinical guideline supported by the Brazilian Sleep Association comprises a brief history of the development of Brazilian sleep physiotherapy, outlines the role of the physiotherapist as part of a sleep health team, and describes the clinical guidelines in respect of the management of some sleep disorders by the physiotherapist (including sleep breathing disorders, i.e., obstructive sleep apnea, central sleep apnea, upper airway resistance syndrome, hypoventilation syndromes and overlap syndrome, and pediatric sleep breathing disorders; sleep bruxism; circadian rhythms disturbances; insomnia; and Willis-Ekbom disease/periodic limb movement disorder. This clinical practice guideline reflects the state of the art at the time of publication and will be reviewed and updated as new information becomes available.

## 1. INTRODUCTION

Almost 10 years after the first Brazilian Consensus on Sleep Physiotherapy^1^, the field of sleep physiotherapy (PT) has changed and improved with advances in many areas due to investigations and research. There is a need to update this knowledge and to create a solid bridge between “the bench and the bedside”, translating into clinical practice the scientific advances. When we understand “where we are” in the field of sleep PT, we can see “where to go”, and the avenues that open to meet the needs of our patients. Sleep PT is still an incipient field worldwide, but is a very promising area. There is already a great deal of teamwork being applied to investigate, create, discover, test, and apply new developments in clinical practice for its unique purpose: to help patients with sleep disorders, including those with a range of comorbid conditions, and improve their quality of life.

The purpose of this consensus is to provide patient-centered clinical guidelines based on a critical analysis of the latest high quality clinical research and the experience of PTs in clinical practice to enable them to make the best decisions in respect of the care of patients with sleep disorders, in addition to describing the area of practice of PT in Brazil. This evidence-based clinical guideline provides a single source of information about the physiotherapeutic management of sleep disorders, integrating contributions from clinical experts, and formulating reliable recommendations for sleep PT practice in Brazil.

The recommendations regarding the physiotherapeutic management of some sleep disorders (obstructive sleep apnea, central sleep apnea, other sleep breathing disorders, i.e., upper airway resistance syndrome, hypoventilation syndromes and overlap syndrome, pediatric sleep breathing disorders, sleep bruxism, disturbances of circadian rhythms, insomnia, and Willis-Ekbom disease/periodic limb movement disorder) were made by subgroups and evaluated together with the task force commission. The approach adopted by the authors included several online meetings with discussions by the different groups of experts in respect of specific sleep disturbances. The discussion was open in nature and driven by the experience and opinions of the participating experts. The task force was formed primarily of 23 sleep PTs involved in teaching, research and clinical practice from a wide cultural and geographical area in Brazil. The literature search strategy was primarily designed to identify meta-analyses and systematic reviews, followed by randomized clinical trials, observational studies, clinical practice guidelines, and case studies. After the literature search, a meeting was held to discuss the evidence identified and the current clinical practice in Brazil carried out according to the relevant laws. Editing of the consensus continued until all authors were in full agreement. The consensus was then presented twice to the task force commission and was open to all authors for discussion. After agreement was reached on the final form and content of the consensus document, which was based not only on a synthesis of the high-quality clinical research, but also on expert opinion, this document was written.

The recommendations of each subgroup were classified according to the Strength of Recommendations Taxonomy (SORT) scale^2^. This scale classifies the level of evidence according to the quality and the consistency of the studies, through an algorithm. The SORT levels of evidence are classified as A, B or C depending on the quality and consistency of the evidence available (Table 1)^2^. In addition to the 3 SORT scale definitions (A, B and C) we added 2 more: “not recommended” and “there is no evidence to support the recommendation of these practices”. The classification of “not recommended” means that there is scientific evidence against the modality, or that in our clinical experience this modality did not present positive outcomes that justify its use/incorporation. The classification “there is no evidence to support the recommendation of these practices” means that we could not even formulate a recommendation, either for or against the modality/intervention due to a lack of literature in respect of the modality and/or a lack of evidence from clinical practice, i.e., scientific and empirical evidence. These classifications should be considered when practitioners are deciding whether to use certain modalities within PT.

**Table 1:** Classification of Strength of Recommendations Taxonomy scale^2^.

Strength of recommendation	Definition
A	Recommendation based on consistent and good quality patient-oriented outcomes.
B	Recommendation based on inconsistent or limited quality patient-oriented outcomes.
C	Recommendation based on consensus, usual practice, opinion, disease-oriented evidence, and case series for studies of diagnosis, treatment, prevention or screening.

We hope that the consistent use of these recommendations will improve the ability and quality of the practice of PTs in the sleep field and help to expand future research to generate new therapeutic options in sleep PT.

## 2. PRINCIPLES OF SLEEP PHYSIOTHERAPY AND ITS LEGAL REGULATION IN BRAZIL

### 2.1. The history of sleep physiotherapy in Brazil

PTs initially had a modest role in the work carried out in sleep research centers and small sleep research groups within intensive care, cardiorespiratory and neurological care groups. In the late-1990s, the use of positive airway pressure (PAP) therapy was incorporated into the treatment of sleep apnea. The demand for PAP devices gradually increased, which helped the growth of sleep PT and its expansion to other aspects of sleep care, not only those related to sleep breathing disorders (SDB).

During this period, the first polysomnography (PSG) course for health professionals took place at the Instituto do Sono in Sao Paulo. The knowledge obtained by the (very few) PTs who attended this course was passed on in their respective workplaces, and thus some physical therapists became early adopters and advocates of the use of PT in the sleep field.

In the field of research, in the 2000s a number of PTs took part in *latu* and *strictu-sensu* postgraduate courses at the Sleep Laboratory of the Heart Institute (InCor), the Neurosurgery Laboratory, and the Pulmonology Department, among other departments at the Faculty of Medicine of the University of São Paulo (USP). The same occurred at the Federal University of São Paulo (UNIFESP), in the Departments of Psychobiology and Neurology/Neurosurgery. Since then, some extension and specialization courses in sleep have also emerged and spread throughout Brazil. Several research groups including PTs were formed in this period, allowing new opportunities for the PT in the field.

Sleep associations contributed to the development of the area of sleep PT. In 2005, the first PT Commission of the Brazilian Sleep Association (ABS) was formed through an initiative with the associated PTs. In 2014, the Brazilian Association of Cardiorespiratory Physiotherapy and Physiotherapy in Intensive Care (ASSOBRAFIR) requested to the Brazilian Federal Council of Physical Therapy (COFFITO) the recognition of PT applied to sleep disorders. In 2021, the ABS in partnership with ASSOBRAFIR, introduced the first certification in sleep PT, with 28 PT from several Brazilian states being certified in respect of their performance and experience in both research and clinical settings^3^.

Through research, teaching and clinical practice, several PT have contributed significantly to clinical practice, including studies on the most effective types of PAP therapy^4-7^, and have collaborated in work to define the guidelines of the American Academy of Sleep Medicine (AASM) regarding the importance of using the nasal mask as the first route of choice in PAP therapy for the treatment of SDB^8^. In 2013, the “Brazilian Consensus on Sleep Physiotherapy” was published^1^. In 2015, one of the first scientific articles on the role of PT in the treatment of SDB was published^9^. Subsequently, other Brazilian studies have emerged covering subjects, as sleep rehabilitation^10^, the timing of rehabilitation in relation to circadian preference, the use of therapeutic exercise^11^, and other PT modalities^12^ as treatments, as well as studies related to pain, an area that has long been known by PT to be influenced by sleep^13^. In parallel with research and clinical activities, since the early 2000s PTs have begun to work in large national and multinational companies that offer products and services in the sleep field.

Thus, the role of PTs in the field of sleep expanded rapidly, working not only in research, clinics, and hospitals but in commercial settings and as consultants. However, there is a lack of sleep PT education, a field that needs to be addressed but is beyond the focus of this consensus.

### 2.2. Legal regulation of sleep physiotherapy in Brazil

Over the years, several PTs were engaged in calling for official recognition of the work of sleep PT. This was accomplished in 2021 by COFFITO Resolution #536^14^, which recognized sleep as an area of work of Brazilian PTs. We highlight the epidemiological, physiological, and pathophysiological knowledge of the PT profession, including evaluation, the adherence, compliance and titration of PAP for SDB treatment, as well as the PT prescription, based on physiotherapeutic diagnosis through the International Classification of Functioning and Health (ICF)^15^, published in 2001 by World Health Organization (WHO).

## 3. THE APPROACH TO THE PATIENT IN SLEEP PHYSIOTHERAPY

There is a consensus that good sleep is essential for good health. Still, there have been few attempts to define exactly what constitutes sleep health^16^. Sleep health is defined as “a multidimensional pattern of sleep-wakefulness, adapted to individual, social and environmental demands, that promotes physical and mental well-being”^16^. This is in line with the definition of health in general produced by the WHO, which is based on positive attributes, rather than simply on a lack of disease^17^. Sleep health is related to individual, social and contextual factors^18^. Increasing evidence demonstrates the association of sleep disorders with other comorbidities and indicates the crucial role of sleep deprivation and/or dysfunction in the development of these diseases^18,19^.

The 3^rd^ International Classification of Sleep Disorders (ICSD-3) describes more than 80 sleep disorders divided into 6 main categories: insomnia, SDB, central disorders of hypersomnolence, circadian rhythm sleep-wake disorders, parasomnias, and sleep-related movement disorders^20^. Obstructive sleep apnea (OSA) is a common sleep disorder, with epidemiological studies indicating a prevalence in adults of between 25 and 46%^21,22^, with the São Paulo sleep study reporting a prevalence of 33%^21^. A population-based study in the city of São Paulo Brazil reported a prevalence of insomnia of 32%^23^. Another very frequent sleep disorder is Willis-Ekbom’s disease (commonly called restless legs syndrome), with a prevalence ranging from 2 to 21% in the world population^24^ and 6.4% in Brazil^25^.

The different sleep disorders can be monitored using the International Statistical Classification of Diseases and Related Health Problems (ICD), which, in its 11^th^ edition, presents a chapter on sleep-wake cycle disorders^26^. ICD can be considered the main coding tool for mortality and morbidity problems^27^. Nevertheless, this information does not express the needs and difficulties that people with different health conditions experience. We suggest that sleep PTs understand and use the International Classification of Functioning, Disability, and Health (ICF), which, like the ICD, is part of the WHO Family of International Classifications. The ICF presents *functioning* as an indicator of health, complementary to mortality and morbidity.

Functioning is the key indicator for rehabilitation^28^ and thus can be considered an important clinical outcome for the PT. In rehabilitation, we seek to restore the functioning of the individual to improve their quality of life and health. For this, the individual is considered in their entirety, relating the problem presented to relevant personal and environmental factors^29^, creating a facilitating physical and social environment, strengthening psychological aspects, and, finally, translating the potential of these improvements into health^28^.

Conceptually, functioning is the generic term for body functions, body structures, activities, and participation, which is influenced by health conditions, environmental and personal factors^30^. The sleep PT must understand the dynamism linked to this concept, since functioning is a *continuum* states that, depending on the influence exerted on its components, can range from full functioning to total disability^31^.

### 3.1. Evaluation of sleep physiotherapy

#### 3.1.1. Main complaint

The evaluation begins with questioning related to the main complaint, which will direct the continuity of the anamnesis, the physical examination, and the subsequent development of the objectives and conduct of the sleep PT. Questions like, “Why are you looking for my help right now?” and “What bothers you most about your sleep?” can help outline the main complaint. Assessing the patient’s perception of the quality of their sleep, and in specific cases (children, dementia syndromes, language impairment, and parasomnias), input from a partner can be significant.

Sleep PTs should be aware that sleep disorders do not only impact the sleep period, but can also have negative daytime consequences, and in different aspects of functioning, (e.g., difficulty driving, focusing on work, or engaging in social activities)^32^. Thus, the main complaint may not necessarily be related to the sleep period itself. Assessing functioning-related problems associated with sleep complaints is, thus, valuable in identifying issues to be worked on during treatment.

When questioning the patient about the main complaint, the sleep PT may come across situations in which the patient reports that they are not the source of the complaint but blame the bed partner. In these cases, to assess whether the patient recognizes, or denies, the possible existence of a sleep disorder will help to identify how ready they are to start PT treatment.

#### 3.1.2. Identification of the motivational stage

**Figure 1:**
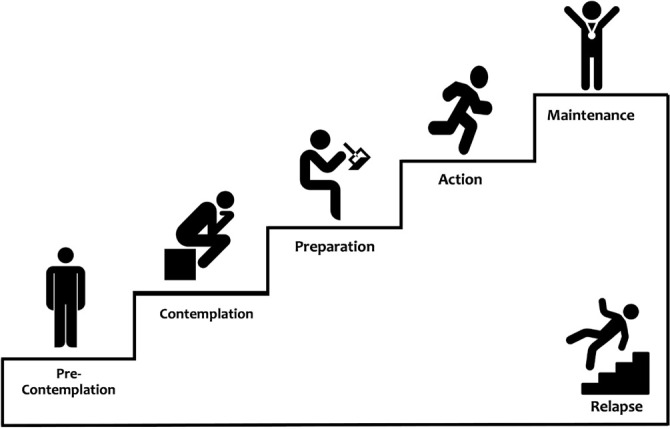
The 5 behavioral stages are according to the Transtheoretical Model of Behavioral Change.

Many interventions proposed by the sleep PT involve the promotion of behavioral changes to increase adherence to the treatment of sleep disorders. Identifying the motivational stage of the patient can help to direct the intervention proposed by the sleep PT. According to the Transtheoretical Model of Behavioral Change, there are 5 behavioral stages: pre-contemplation, contemplation, preparation, action, and maintenance^33^ (Figure 1).

In the pre-contemplation stage, the patient denies the existence of the problem and is reluctant to consider what needs to change in their habits. At this time, it is relevant to question the patient to increase their perception of the problem.

In the contemplation stage, the patient begins to realize that they have a problem, but fear and insecurity prevent them from acting. In this stage, the patient tends to be very defensive and justifies their position, when deep down they would like to start the process of change. In this period of ambivalence, the PT needs to draw the patient’s attention to the risks associated with not changing their behavior and encourage them to believe in the possibility of change.

In the preparation stage, the patient is beginning to understand and realize how some changes can be beneficial. The role of the PT is to guide the patient in respect of the most appropriate way to get the changes they desire so that they can then move to the next stage, the action.

In the action stage, the patient takes the first steps to modify their behavior and begins to make some changes. The PT should facilitate this process by helping the patient to make this a habit.

The fifth and final stage is maintenance, in which discipline is necessary to avoid relapses. The sleep PT will help in building strategies to maintain the target behavior and overcome the factors that can threaten this.

In the Transtheoretical Model of Behavioral Change the individual does not necessarily progress through the stages in a precise linear way, but can move forward or back through the stages before reaching their ultimate goal^33^. The assessment of the motivational stages should be made by the sleep PT listening carefully to the patient, with the patient activating their motivation for change and the consequent adherence to treatment.

#### 3.1.3. History of current and previous disease

In the development of the history of the current disease, the sleep PT should explore the process that led to the main complaint chronologically and seek to identify the factors that aggravate or relieve the condition.

In the previous history, the presence of neurological, cardiac, pulmonary, otorhinolaryngologic, and psychiatric diseases should be noted and their relationship with the main complaint should be considered. Conditions, such as chronic pain, dementia, asthma, heart failure, depression, and anxiety disorders are often observed when dealing with complaints of insomnia; patients with hypothyroidism, obesity, and inflammatory diseases often complain of excessive sleepiness; anemia, kidney disease, and pregnancy can cause or exacerbate Willis-Ekbom disease; cough, choking, heartburn and gastric reflux, as well as changes in libido and sexual impotence, may be associated with SDB^34^.

Seeking information about cognitive functions, (i.e., lack of concentration, attention and memory), can be complaints associated with poor sleep quality or reduced sleep duration, as can complaints related to mood. Excessive sleepiness, fatigue, restless sleep, dry mouth upon awakening, and headache are symptoms that need to be evaluated and may be associated with different sleep disorders^34^.

When investigating obstructive types of SDB, ask about previous surgical procedures, especially nasal and upper airway (UA) surgeries. Information about current or previous smoking should be considered as nicotine dependence can be associated with a range of sleep disorders^35^.

As for the sleep routine, an interesting approach is to ask the patient to describe their sleep routine, specifying the time they go to bed, go to sleep, wake, and get up; the regularity of these times; the maintenance of these schedules on weekends and activities carried out before bed (reading, watching television or activities involving screens/light emission). Observations of the patient’s satisfaction in respect of their sleep schedules, sleep latency, and sleep fragmentation are warranted. Individuals with insomnia often report inadequate nighttime sleep and may have difficulty in respect of sleep onset, maintaining sleep, waking up too early, or returning to sleep (more details in the Section 11). In patients with SDB, for example, it is common the complain of difficulties in maintaining sleep, but these patients usually have a lower sleep onset latency due to excessive sleepiness^34^. Among the reasons that lead to awakenings, nocturia, characterized by the presence of at least 2 arousals to urinate, is an aspect to evaluate and may be associated with SDB^36^. Information related to night shift work, and sleep time preferences need to be investigated^34^.

The patient should be questioned in respect of their sleeping environment to evaluate whether it is optimum to promote sleep; the essential aspects of the evaluation are luminosity, the presence of noise, temperature, the presence of bed partners and/or pets, the activities performed and the characteristics of the bed. The ideal environment should be dark, quiet, thermally pleasant, and used only for sleeping and having sex.

Sleep ergonomics should be evaluated as the choice of the sleeping position may be related to SDB and pain conditions that can lead to sleep fragmentation.

The evaluation of the sleep PT should also cover the presence of other comorbid sleep disorders, as hypersomnias, parasomnias, other sleep respiratory disorders, and circadian rhythm and movement disorders related to sleep. If the patient sleeps accompanied, the reports of the partner, including the presence of snoring, breathing pauses, grinding of the teeth, somniloquy, or excessive movements in bed are useful. It is helpful to obtain the patient’s report about their perception of the quality of their sleep. The assessment of psychosocial, occupational, academic, and physical activity, as well as satisfaction with personal relationships can provide valuable information about the impact of sleep disorders on the patient’s life.

Table 2 describes the aspects related to sleep that should be investigated during the PT evaluation, and which should be directed according to the patient’s main complaint and clinical history.

**Table 2:** Aspects related to sleep to be investigated during the physiotherapeutic evaluation.

The routine of sleep: ▪ Regular times to sleep and wake up ▪ Sleep onset latency ▪ Duration of sleep ▪ Routine maintenance on weekends ▪ Daytime naps	On the environment: ▪ Is your room cozy, and comfortable? ▪ Is your room noisy? ▪ Is the room temperature comfortable? ▪ Are there other people or pets in the same room? ▪ What activities do you do in the bedroom besides sleeping?
**Sleep hygiene:** ▪ Do you watch TV in bed? ▪ Do you lie in bed when you cannot sleep? ▪ Do you read in bed? ▪ Do you use a smartphone in bed? ▪ Do you smoke at night? ▪ Do you consume alcohol or caffeinated drinks before bed? ▪ Do you consume heavy meals in the evening? ▪ Do you undertake a physical exercise in the evening?	**On socioeconomic conditions:** ▪ Social and financial problems ▪ Access to health services
**Sleep fragmentation:** ▪ How often do you wake up in your sleep? ▪ What are the reasons? ▪ How long does it take to return to sleep? ▪ Do you stay in bed when you can not sleep? ▪ How often do you go to the bathroom to urinate during the night?	**Morning symptoms:** ▪ Restful sleep repair ▪ Excessive drowsiness ▪ Dry mouth on awakening ▪ Head pain ▪ Congestion ▪ Reflux or heartburn
**During sleep:** ▪ Do you experience choking or a sensation of suffocation? ▪ Do you cough? ▪ Do you have reflux? ▪ Do you sweat? ▪ What is your preferred sleep position? ▪ Do you grind your teeth during sleep? ▪ Do you have a sensation of tension or stiffness in the muscles of the face? ▪ Have you experienced apnea? ▪ Can you hear loud snoring from the next room? ▪ Do you have aggressive movements during sleep? ▪ Do you speak in your sleep? ▪ Do you have nightmares? ▪ Do you sleepwalk? ▪ Do you act out your dreams? ▪ Do you move your limbs? ▪ Do you have cramps?	**Day functions:** ▪ Drowsiness and/or accidents caused by drowsiness ▪ Tiredness ▪ Concentration deficit ▪ Memory deficit ▪ Fatigue ▪ Irritability ▪ Pain
**Others:** ▪ Sexual dysfunction ▪ Weight changes ▪ Medicines and other substances in use ▪ Comorbidities ▪ Previous surgeries ▪ Unpleasant sensations in the legs, especially at night, late in the day, or when sitting at rest ▪ Sensation of tension or stiffness in the muscles of the face ▪ Shift work	

Notes:

* = Indicates that there is no specific cutoff point and that data should be evaluated clinically in conjunction with anamnesis and physical examination; “-” = Does not apply due to heterogeneity and subjectivity in completing the sleep diary; OSA = Obstructive sleep apnea; SDB = Sleep breathing disorders; Sens. = Sensitivity; Spec. = Specificity; The questionnaires and scales can be used before and after the PT intervention, to compare the effectiveness of PT and/or rehabilitation.

### 3.2. Knowing the patient: contextual information

#### 3.2.1. Age and sex

The age of the patient is essential information in the evaluation performed by the sleep PT. Quantity and distribution of sleep stages are usually different as age groups change^37^. The prevalence of some sleep disorders changes according to age and sex, as well as their etiological factors^21,38^. The functioning is directly influenced by these individual characteristics.

#### 3.2.2. Work and family context

The involvement (or not) in work activities can impact the habits and routines of the patient and, in turn, influences the sleep routine. There is scientific evidence that social support can influence adherence to treatment^39,40^. The sleep PT should collect information about the family context, whether the patient sleeps accompanied in the same room and whether they live with their children, among other factors. This enables the PT to identify whether the family acts as a barrier or as a facilitator to the treatment, and then to include the family in the educational sessions to adjust their behaviors to provide better adherence.

#### 3.2.3. Eating habits and physical activity

Eating habits and physical activity play important roles as synchronizers of the circadian rhythm. Information on alcohol and caffeine consumption, their amounts and schedules are needed since these substances have a direct effect on sleep patterns and quality^34^. A conversation about eating habits and mealtimes can reveal valuable information about the general state of health of the patient. Similarly, questioning the frequency and intensity of physical activity and their schedules can help the sleep PT better understand the patient’s habits.

#### 3.2.4. Medications in use

Although the sleep PT does not intervene in the prescription of medications, knowledge about the drugs used by the patient is fundamental, including herbal medicines and dietary supplements. Special attention should be paid to medications and substances used to change the waking-sleep cycle. Drugs may have adverse effects that promote sedation or wakefulness. Understanding medications and their effects help the sleep PT to have a global view of the patient’s health, as well as better understand their signs and symptoms, which may or may not be related to sleep disorders and/or their therapeutic behaviors. Sleep PT is part of an interdisciplinary team and can refer the patient to a specialist whenever necessary.

### 3.3. Physical examination

#### 3.3.1. Vital signs

It is suggested that the sleep PT starts the physical exam by measuring pulmonary auscultation, peripheral oxygen saturation, and heart rate during waking at rest.

#### 3.3.2. Anthropometric assessment

The assessment of weight, height, and body mass index provides essential information for the sleep PT. Some sleep disorders are directly related to being overweight and, in addition, changes in these aspects over time may require changes in behavior.

It is suggested that the sleep PT evaluate the neck circumference, especially in cases of suspected OSA. Neck circumference varies between genders^41^. In an epidemiological investigation in Brazil, the cutoff point for mild to severe OSA for men was 40.2cm (accuracy 70%) and in women 36.2cm (accuracy 76%)^42^. Other measures to consider include abdomen circumference and waist-to-hip ratio as they reflect body fat distribution and cardiovascular risk. The cut points for waist circumference are >102cm for men and >88cm for women in respect of identifying those with increased cardiovascular risk^43^.

#### 3.3.3. Inspection and palpation of craniofacial and neck structures

The evaluation of craniofacial structure is significant, especially when there is suspicion of SDB^44^. Characteristics such as a long or short face; the size, proportions, and positioning of the maxilla and mandible, as well as the shape of the palate and the volume of the intraoral structures (i.e., tongue, uvula, and soft palate) help to identify risk factors for OSA. Modified Mallampati classification or Friedman tongue position classification are used for evaluation of the oropharynx region^45^.

Regarding SDB, the nasal cavity requires special attention. It is suggested that the sleep PT asks the patient about their preference for the nasal or oral route of breathing, both during wakefulness and during sleep. In addition, they should ask about nasal dryness and the oral cavity. It is suggested that the PT evaluate the patient’s nose about its size, shape, and possible deviations that can be identified externally.

Regarding sleep bruxism, the evaluation of craniofacial structure associated with the evaluation of the neck and thoracic spine, and upper limbs are essential for treatment. It may be necessary for the PT to refer the patient to a dentist. It is up to the PT to recognize changes in function, in respect of muscle activity; movement of the temporomandibular joint (TMJ); reduced range of movement in the TMJ, mobility, and muscle strength, including in antagonistic and synergistic muscles related to the movement of the TMJ; positioning at rest and at movement of the TMJ (more details in the Section 9).

#### 3.3.4. Inspection and palpation of other structures

The assessment of the spine and its curvature may be necessary (some scoliosis may compromise ventilation or contribute to chronic pain that may interfere with positioning during sleep). The evaluation of edema in the lower limbs is of paramount importance for SDB, to control and/or treat the rostral displacement of fluids during the recumbent position.

In pain conditions, a pain map, in which the patient colors/shades the pain sites, as well as the visual or numerical pain scale, can be used. Although pain is a personal and subjective experience, the use of these instruments can help to understand the intensity of and the evolution of pain during treatment^46^.

### 3.4. Questionnaires and scales: subjective evaluation

The sleep PT should know the main assessment tools used for the screening and clinical follow-up of patients with sleep disorders. Among the questionnaires and scales described in the literature, some of them are disease-specific, relating to factors (e.g., drowsiness or the presence of awakenings), while others evaluate sleep in a more general way, especially in respect of sleep quality or circadian preference. Table 3 summarizes the self-administered questionnaires translated, validated, and culturally adapted for the Brazilian population.

**Table 3:** Questionnaires and scales for the evaluation of sleep disorders and/or conditions were translated into Portuguese and adapted, and culturally validated for use in Brazil.

Condition	Assessment	Instrument	Construct	Outcomes	Psychometric properties sensitivity/specificity
**Sleep breathing disorders**	OSA screening	Berlin questionnaire^47^	A simple self-administered questionnaire used to identify and to predict the risk of OSA Consists of 10 items distributed in 3 categories: 1 - apnea and snoring; 2 - drowsiness, and 3 - presence or absence of obesity and history of hypertension	≥2 completed categories, high risk for OSA	**AHI>15/h** Sens.: 86.2% Spec.: 54.7% **AHI>30/h** Sens.: 93.8% Spec.: 50%
	OSA screening	STOP-Bang^48^	Composed of 8 questions with “Yes” or “No” answers, which address items related to the individual’s anthropometry and the presented symptomatology	≥3, high risk for OSA	**Mod. and severe OSA** Sens.: 88.6% Spec.: 35.2%
	OSA screening	NoSAS Score^49^	Simple and effective screening tool for individuals with suspected OSA Scores range from 0 to 17, addressing items related to the individual’s anthropometry, presence of snoring, age, and gender	>8, high risk for OSA	Sens.: 85% Spec.: 77%
**Pain and sleep**	Identification and prediction of the risk of the sleep-pain association	Sleep assessment instrument for the elderly with pain^50^	Practical and comprehensive instrument to assess the co-occurrence of chronic pain conditions and sleep disorders in the elderly Composed of 7 items with “Yes” or “No” responses, grouped according to the sleep dimensions: sleep latency, sleep maintenance, physical discomfort (tiredness, exhaustion, and fatigue), self-perception of sleep, daytime sleepiness, sleeping medications	^*^	Sens.: 73.2% Spec.: 79.1%
**Sleep quality**	Sleep quality	Pittsburgh sleep quality index^51^	Evaluates the quality of sleep in the last month Composed of 19 questions, categorized into 7 components (subjective sleep quality, sleep latency, sleep duration, sleep efficiency, sleep disorders, use of sleeping medication, and sleepiness and daytime dysfunctions) The total score ranges from 0 to 21	≥5, poor sleep quality <5, good sleep quality	Sens.: 80.0% Spec.: 68.8%
	Sleep quality	Mini sleep questionnaire^52^	Composed of 10 questions, evaluates the frequency of sleep-related complaints	10-24, good sleep 25-27, slightly altered sleep 28-30, moderately altered sleep >30, very altered sleep	--
**Circadian preference**	Circadian preference	Morningness-eveningness questionnaire^53^	Identifies the circadian preference of respondents, and classifies as extreme eveningness(or extreme evening-type), eveningness (or evening-type), indifferent, morningness (or morning-type), or extreme morningness (or extreme morning-type)	16-30, extreme eveningness 31-41, eveningness 42-58, indifferent 59-69, morningness 70-86, extreme morningness	--
**Sleepiness**	Excessive daytime sleepiness	Epworth sleepiness scale^54^	Evaluates the probability of falling asleep in 8 situations involving monotonous daily activities	0-10, normal >11, excessive daytime sleepiness	Sens.: 45% Spec.: 81%
**Willis-Ekbom disease/Restless legs syndrome**	Disease severity	International restless legs syndrome study group rating scale^55^	Evaluates the severity and impact of the disease on the patient’s life Composed of 10 Likert-type questions (0 to 4) The questions refer to 1 week and access the symptoms and their frequencies, as well as their impacts on the respondent’s life The score varies between 0-40 points	0-10, mild 11-20, moderate 21-30, severe 31-40, very severe	Reliability of 80%
**Insomnia**	Severity of insomnia	Insomnia severity index^56^	Evaluates the severity of insomnia. Composed of 7 questions that assess parameters associated with insomnia in the last 2 weeks The score varies from 0 to 4 points for each question to measure sleep latency, sleep maintenance, early awakenings, sleep satisfaction, interference in daytime functioning, and the level of sleep stress The total score is from 0 to 28 points	0-7, absence of insomnia 8-14, subliminal insomnia 15-21, moderate insomnia 22-28, severe insomnia	--
**Pediatric population**	Sleep conditions	Sleep disturbance scale for children^57^	Evaluates sleep among children aged 3-18 years Differentiates conditions (i.e., disorders of initiating and maintaining sleep, SDB, disorders of arousal, sleep-wake transition disorders, excessive somnolence, and sleep hyperhidrosis Composed of 26 questions with 5 response answer	--	Reliability >55%
	Excessive daytime sleepiness	Pediatric daytime sleepiness scale^58^	Evaluates the occurrence of excessive daytime sleepiness 8 multiple choice questions Each question has 5 response options, using a Likert scale: 0 = never; 1 = almost never; 2 = sometimes; 3 = frequently, and 4 = always; The total score is from 0 to 32 points	Higher scores indicating more sleepiness	Reliability of 78%

Some measurement instruments have been translated unofficially. Although they are used in clinical practice and research, they lack specificity and sensitivity because they have not been validated. These instruments include the Stanford sleepiness scale, and sleep diaries. The latter is used concomitantly with the use of actigraphy and is important in the evaluation of the sleep-wake pattern through recording the time to go to bed, sleep, wake up, night awakenings, and daytime naps. This allows the analysis of routine and habits related to pre-and post-sleep using subjective data gathered over an extended period^59^.

### 3.5. Interpretation of sleep tests: objective evaluation

The sleep PT should have extensive knowledge of the diagnostic methods available. Each method has its particularities, limitations, and specific indications and can help in the physiotherapeutic evaluation.

The type I sleep study, also known as type I PSG, or complete polysomnography, among other names, is considered the most complete way to evaluate the various variables that affect human sleep. It comprises an electroencephalogram, an electrooculogram, an electromyogram of the chin and tibial anterior muscle, an electrocardiogram, monitoring of airflow channels, respiratory effort sensors, oximetry, audio/video recording, position and snoring sensors. PSG is performed with the supervision of a PSG technician trained to identify potential artifacts and reposition sensors when necessary^60^. It is widely used in clinical practice and scientific research and is considered the gold standard for the nosological diagnosis of SDB, REM behavior disorder, and periodic limbs movement disorder. PSG performed in the sleep lab can provide split-night tests, with the initial portion being used for diagnostic purposes and the final portion for positive pressure titration.

The type II sleep study, known as in-home PSG, records the same variables as type I studies, with the main difference being that it is not performed in a sleep lab, and there is no supervision by a PSG technician. This type of study can be performed in a home environment, in a hospital, or in another environment. The main advantages associated with this method are the possibility of examining the patient’s usual sleep environment, and that it can be applied to patients with mobility restrictions who are unable to travel to a sleep laboratory. This method is subject to a greater number of artifacts due to the absence of a trained professional who can ensure the technical quality of the record. Taking this into account, the analysis of the report, which is composed of the same information of type I tests, should be done with care.

The type III study, known as respiratory polygraphy or home sleep apnea test, aims to evaluate the presence of OSA in patients at a clinical evaluation and is used in association with OSA risk stratification questionnaires. Composed only of nasal airflow signal, a respiratory effort sensor, oximetry, and sometimes a position sensor, this method is normally performed in the patient’s sleep environment. The practicality and greater comfort of this method may be offset to some extent due to its limitations, especially in respect of the absence of channels that assess the presence of sleep and its fragmentation, preventing the marking of respiratory effort related arousal (RERA) and hypopnea validated by arousal. The information available in the report are a respiratory event index (REI), the oxyhemoglobin desaturation index (ODI), and data related to the differentiation of the type and origin of events and body position, which should be interpreted carefully considering the limitations described. This method is not indicated for patients who, beyond the suspicion of OSA, have comorbidities or other associated sleep disorders^61^.

The type IV study, which is used as a screening tool for OSA, it comprises an oximetry record, heart rate and sometime airflow. Studies show a good correlation between ODI obtained by this method and the apnea and hypopnea index (AHI)^62^. Generally, the simplicity of the method means that it does not include relevant information, data on sleep and respiratory events.

Peripheral arterial tonometry evaluates arterial tone via peripheral sensors and detects changes in heart rate and desaturations associated with the end of respiratory events and can estimate the AHI^63^.

Actigraphy is an examination indicated to assess sleep/wake patterns in individuals with suspected circadian rhythm disorders and insomnia. The actigraphy estimates sleep using an accelerometer that detects the increase or reduction of activity (movement). This method can be used in a complementary way to simpler methods of evaluation of OSA, such as the type III and IV exams, which alone do not evaluate sleep variables^64^.

Finally, sleep endoscopy is an examination performed during drug-induced sleep to visualize the point of collapse of the UA. Sleep endoscopy can help in the investigation of possible causes that lead patients with OSA not to adapt to PAP therapy through the documentation of anatomical factors that impact adherence to PAP therapy^65^. However, because it is an invasive method and involves specialized medical training, its clinical applicability is limited to the evaluation of patients with OSA indicated for surgical interventions and in clinical research^66^.

When interpreting the results of these different examinations, the sleep PT needs to carefully consider the limitations of each method. Their knowledge about the sleep habits of the patient and the way the examination was conducted, and, in the case of PSG, whether the night in the sleep laboratory reflected a normal night’s sleep, should be taken into account when interpreting the information gathered. When there is a suspicion of respiratory disorders, the sleep PT must analyze variables, (e.g., AHI, RDI, REI, and ODI), the type of respiratory events (apnea *versus* hypopnea *versus* RERAs), the origin of the events (obstructive *versus* mixed *versus* central), the duration of the events, the association with desaturations and/or awakening, the relationship of the events with the body position adopted during sleep and the distribution of respiratory events at different stages of sleep (NREM *versus* REM). The analysis of this information is essential for the sleep PT to understand the potential phenotypes and endotypes associated with the respiratory disorder, and be able to establish the best treatment plan to restore patient functioning.

In addition to the descriptive and numerical variables, the production and interpretation of hypnograms (graphs representing the stages of sleep) and other graphical representations of the patient’s sleep can not only assist the sleep PT to understand the data but can be used to facilitate the process of education and awareness of the patient about the sleep disorder.

If necessary, the analysis of complementary tests such as blood gas and pulmonary function can help PTs to better understand the SDB that affects the patient. Although patients may have the same sleep disorder, the effects presented may be unique for each individual. A properly conducted evaluation process will allow the sleep PT to generate a significant amount of information regarding impairments in each functioning domain in respect of body function and structures, limitations in activity, and restrictions in participation - always considering the context in which the patient is inserted. It is not the nosological diagnosis that should be considered as the basis for the treatment of the patient’s problem, but the physiotherapeutic diagnosis based on the impact of the condition on the patient’s disability. After completing the evaluation, the sleep PT should use the collected data to establish specific goals and a therapeutic plan personalized as far as possible to meet the needs of each patient. The multidimensionality of sleep disorders and their relationships with so many concomitant variables can often require the involvement of other professionals from the transdisciplinary team.

## 4. PROFESSIONAL INTEGRATION IN SLEEP: A VISION OF PHYSIOTHERAPY

The sleep field is quite challenging due to the multidimensionality of the factors that contribute to the onset or persistence of sleep disorders. Sleep disorders have a multifactorial origin and occur concomitantly with other clinical conditions, compromising patient adherence to appropriate treatment^67^. Integrating the knowledge of professionals from different areas/disciplines would seem to be the natural choice in the context of sleep to create a team that can relate in a multi, inter or transdisciplinary way^67-69^. The PT is a professional who can play a significant role in this team, working both in the prevention and treatment of different sleep disorders^70^.

In the multidisciplinary team, the patient receives independent assistance from professionals, and work in a non-integrative way without the knowledge being exchanged^71^. The specialists share a common objective, but act within their own disciplines. Thus, situations may occur in which the performance of each independent professional may not advance^71^. When professionals place themselves at the margin of their own fields to develop new concepts and ideas, an interdisciplinary team can be created. Interdisciplinary working occurs when 2 or more disciplines are synthesized, establishing a new level of conversation/discourse and integration of knowledge^71^. The transdisciplinarity concept emerges from integration, in which specialists share their roles, helping others to acquire skills related to their area of expertise. This does not simply mean the acceptance that the other can play the role that a particular specialist has been able to do. On the contrary, it is interpreted as expanding the role of the specialist beyond what they were trained to do^72^.

### 4.1. The integration of knowledge for the definition of therapeutic goals

**Figure 2:**
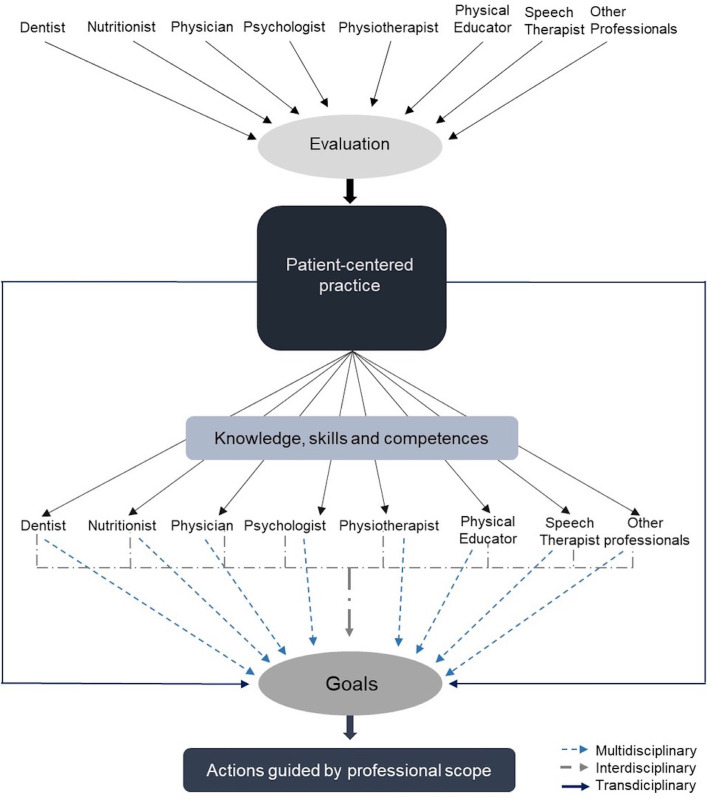
The integration of knowledge to define therapeutic goals in the transdisciplinary approach to the patient.

In the multidisciplinary team, the different professionals implement their actions to individually achieve the goals. In the interdisciplinary team, the goals of the team are first agreed among the members, and each professional makes their contribution to the common plan of joint action. In the transdisciplinary team, not only the goals but the skills are shared^73^ (Figure 2). The assessments of all health professionals involved in the case must be considered, as they are complementary and based on their respective scopes of clinical practice.

In patient-centered care, it is recommended that the established goals should be aligned with the patient’s goals. It is necessary to identify the goals and objectives of the patient during the initial approach, aiming to maximize the results of interventions^74^ (Figure 2), giving the patient the capacity to self-manage his/her health condition^75^.

#### 4.1.1. Knowledge of the skills and competencies of the different team professionals

Regardless of how the team integrates (multi, inter or transdisciplinary), there is a need of a common goal and a shared vision among the members, understanding and respecting the fundamental principles and concepts of each discipline/faculty^72^. The communication resources currently available are facilitators so that this service model can be feasible even among professionals from different cities or states through interprofessional networking^75^.

### 4.2. Professional integration in the clinical context

Through the integration of knowledge, complex problems can be solved and different perspectives can be brought to bear on the same problem^71^. Effective teamwork increases the learning and development of people and organizations, allows better use of resources and implementation of plans, minimizes unnecessary spending, and improves performance and the quality of work^76^.

Among professionals working in sleep, integrative collaboration should be used to deliver both curative or restorative therapy, with strategies directed toward disease prevention, health promotion and improved well-being. PTs must have the knowledge and skills to promote healthy sleep habits at the primary health care level to promote healthy sleep patterns in the general population, even among those without a diagnosis of sleep disorder or other specific disease^77^.

#### 4.2.1. Goals of professional integration in sleep

Given the evident negative impacts of sleep disorders on the general health and well-being of individuals, there is currently a movement among health professionals in Brazil to expand the screening of patients with signs and symptoms indicative of a sleep disorder. Despite considerable advances in recent decades that have allowed us to understand the complexity of sleep disorders, many patients with sleep problems remain underdiagnosed. Different co-existing sleep disorders in the same patient have also been undertreated, despite their considerable frequency^78^. Given this scenario, a teamwork model with individualized diagnosis, risk stratification and treatment are essential in the treatment of different sleep disorders, either when they occur alone or in co-occurrence, leading to potential benefits to the patient^79^. This scenario points out to the importance and evidence of sleep aspects in positive outcomes for health^80,81^, it is imperative to insert the discipline of sleep in the basic curricula of the undergraduate courses of PT, to expand the professional knowledge in approaching sleep in clinical daily practice.

### 4.3. The patient as the main beneficiary of professional integration

Professional integration is associated with improved results - including greater diagnostic accuracy, an improvement in treatment quality and a reduction in individual and social costs related to different diseases^82,83^. There is a lack of evidence about the effectiveness of this approach in the area of sleep, with the little evidence that there is being mostly related to SDB^69,84,85^. In the treatment of sleep problems in general, empirically, there is a noticeably greater engagement of patients and a consequent improvement in results using this method. There is an urgent need to strengthen the scientific evidence in respect of the effectiveness of team care and its ability to produce better clinical outcomes, as well as to confront the idea that this approach raises health care costs without adding greater benefits.

### 4.4. The physiotherapist in the sleep team

PTs are promoters of healthy behaviors and good health^86-89^. As PTs are rehabilitation professionals, the main outcome of the treatment provided by PTs is improved functionality^28^. The scope of PT practice includes the screening and treatment of sleep issues that have a direct impact on patient functionality. PTs are in an ideal position to promote health and well-being to their patients through improved sleep^86^. PTs have expertise in non-pharmacological and noninvasive interventions, educational pathophysiology baggage, as well as knowledge and skills related to well-being and therapeutic exercise. PTs often have the opportunity to spend more time with the patient because of the nature of the treatment, which frequently allows a relationship of trust with the patient to be developed more quickly. These attributes are crucial in the context of chronic non-communicable diseases, regarding prevention (reducing risk factors), reversal and management^90^, with actions aligned with the biopsychosocial care model^86^.

It is paramount to develop processes that facilitate the individualized treatment of every patient through the engagement of a team of professionals. These teams should be developed by encouraging communication between different specialists, and by all members of the team showing mutual respect for the capacities, competencies, responsibilities, and clinical scope of each member, with the integration and involvement of the patient in the treatment as a key element in the therapeutic process.

## 5. OBSTRUCTIVE SLEEP APNEA IN ADULTS

**Figure 3:**
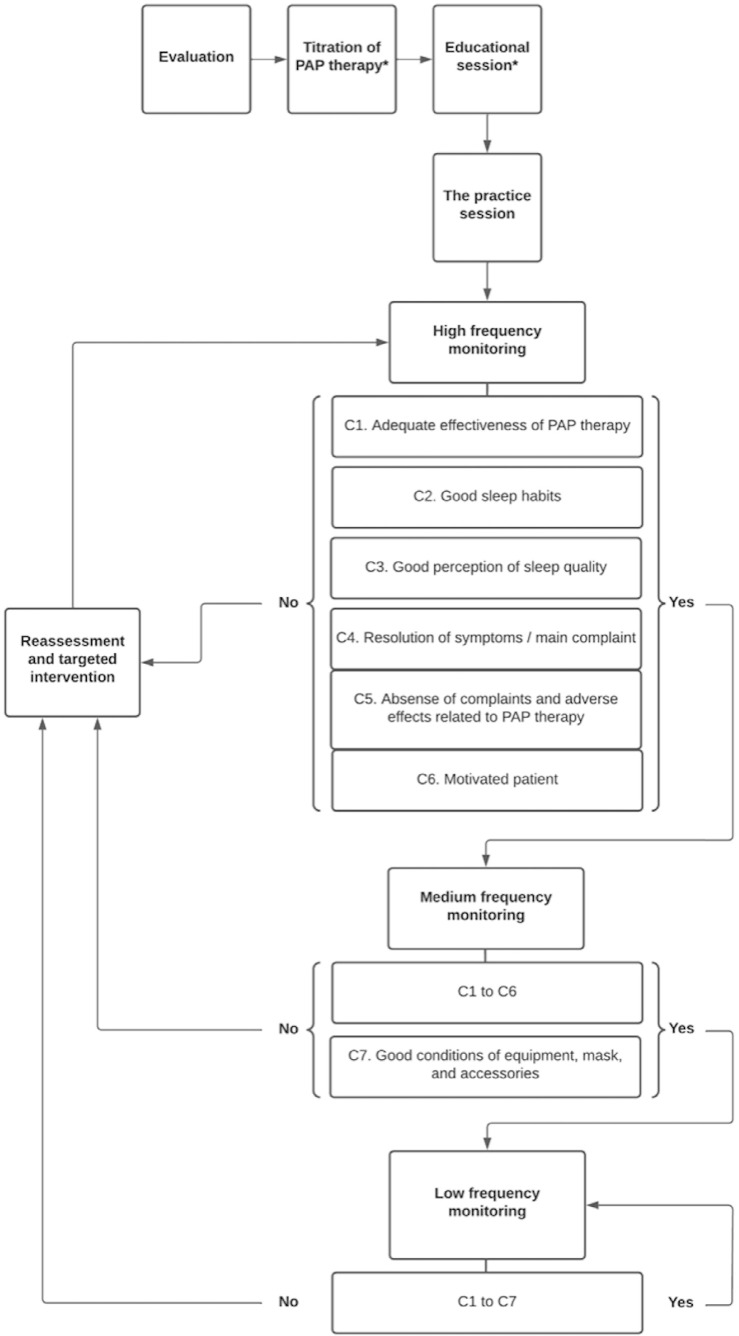
Flowchart illustrating a suggested protocol for the treatment of OSA using PAP therapy by sleep PTs.

Notes: *Assess the need for a new PAP therapy titration and/or educational session at all stages. C = Criterion; PAP = Positive airway pressure.

Sleep PTs play a key role in the process of adaptation to and management of positive airway pressure (PAP) therapy for the treatment of OSA. It is crucial the interaction with the medical team for receiving supportive information about the proposed treatment. Figure 3 illustrates a suggestion for the management of the physiotherapeutic treatment of OSA. In addition to PAP therapy, the sleep PT can contribute to the treatment of OSA through therapeutic exercises, respiratory muscle training, education, and the promotion of strategies for good sleep health.

### 5.1. Physiotherapeutic objectives

#### Promote good habits related to sleep;

- Resolve the main complaint and other symptoms related to sleep;
- Ensure good efficacy of and adherence to PAP therapy and/or therapeutic exercises and/or respiratory muscle training;
- Eliminate the possible adverse effects related to PAP therapy;
- Motivate the patient to improve their sleep;
- Improve sleep quality;
- Improve quality of life;
- Improve aspects of functioning.

### 5.2. Role of the physical therapist

#### 5.2.1. Physiotherapeutic evaluation of OSA

The physiotherapeutic assessment of patients with OSA (described in more detail in the Section 3) comprises behavioral assessment, physical examination, the application of specific screening tools, sleep studies and titration. The evaluation should aim for a physiotherapeutic diagnosis, particularly in respect of factors that will improve the better management and treatment of OSA.

5.2.1.1. Assessment of behavior

The beginning of PAP therapy involves a process of behavioral change. Understanding the expectations and motivational stage of patients with OSA can help the sleep PT in their actions^91.^

Identification of the motivational stage

For the recognition of the motivational stage, the Transtheoretical Model of Behavioral Change (more details in the Section 3) divides change into 5 behavioral stages^33,92^. In the first stage of pre-contemplation, the most pertinent aspect is the awareness of the consequences of not treating OSA^93^. In the second stage, contemplation, it is up to the sleep PT to assist in motivational questions in an individual way and directed to the interests of each one, with open questions related to the pros and cons formulated by the patient himself (Table 4)^91^.

**Table 4:** Examples of cons and pros sentences that can be formulated by the patient and the PT respectively in the contemplation stage.

	Sentence examples
CONS	The mask is uncomfortable; My partner won’t find me attractive; The device is very large; I will have to take it everywhere I go to sleep; Will it make a noise.
PROS	There are several models of mask made with light material that are easy to put on and take off; You will have more energy to spend with your partner, because you feel better; The device is lightweight, easy to carry; The devices are very quiet; You can be more productive at work; Your blood pressure may become more controlled; You will have a lower risk of developing other diseases.

In the third stage of preparation, the support of family and friends, and particularly of partners can be used to motivate the patient (extrinsic motivation) in respect of the treatment. The next stage, that of action, is the phase in which goals must be drawn. As the goals are reached, skills will be built, and the effectiveness of the treatment will generate increasing self-confidence and motivation (intrinsic motivation). Once the patient is comfortable using PAP, they enter the maintenance stage, and the PT must work to create sustained behavioral change and prevent relapse^91^. The use of telemonitoring, with calls and messages of motivational content, can help at this stage.

Motivational interview

Motivational interviewing is a person-centered guiding process and is used to help individuals change their behavior or learn new skills. The approach aims to help people talk and resolve their ambivalence towards behavioral change, using their own motivation, energy and commitment^94^. A skilled PT is able to alternate between the styles of directing, guiding and monitoring in response to the needs of the patient. This requires the use of 3 basic skills: asking, listening and informing^94^.

5.2.1.2. Physical examination

A physical examination (more details in the Section 3) is fundamental for patients with indication of PAP therapy, mainly to determine factors that will affect the choice of interface.

5.2.1.3. Polysomnography

For the diagnosis of OSA, we can use polysomnography (type I and II) and the home sleep apnea test (type III). It is essential that the sleep PT can judge the reliability of the report presented. This depends on the examination being performed under the usual conditions of the patient in respect of bedtime and waking up time, the position adopted for sleep, the use of medications and alcohol consumption, among other factors.

The results of type I and II PSG should be carefully analyzed, with the extraction of information describing sleep variables and variables related to breathing disorders^95^. This analysis will allow the sleep PT to understand the disorder that affects the patient and draw up the best therapeutic plan for restoring the affected functioning.

The sleep PT should understand the limitations of type III studies (respiratory polygraphy) and refer any patient with clinical suspicion of OSA who presents a negative result in this type of exam to a type I sleep study^61^. Type IV study basically comprises night oximetry and has been used by PTs only as a screening tool and for the evaluation of the effectiveness of the proposed treatments, in addition to being used to improve home titration.

5.2.1.4. Titration of PAP therapy

The objective of titration is to determine the lowest pressure that is able to eliminate respiratory events during sleep^96^.

Manual titration in a laboratory

Manual titration in the laboratory is the gold standard to determine the optimal treatment pressure for patients with OSA, and is indicated in cases with severe comorbidities, for patients who use drugs that depress the respiratory center, have other sleep disorders and in cases of Bilevel PAP (Bilevel) titration. In laboratory, continuous PAP (CPAP) and Bilevel PAP (Bilevel) titration is often performed by the sleep technician. The PT must know the advantages and disadvantages of this method. The main advantage is the real-time monitoring of the recording channels, enabling the immediate solution of problems that may prevent the titration of optimal pressure which may affect the patient’s adherence to the treatment^96^. Disadvantages include recording only one night’s sleep, that the patient may have difficulty falling asleep in a monitored environment while coupled to sensors and factors that may interfere with the time required for optimal pressure titration such as sleep quantity, proportion of REM sleep, and proportion of time in the dorsal decubitus position^97^. In Table 5, we present the criteria for grading manual titration.

**Table 5:** Criteria for manual PAP titration grading according to the AASM Task Force^96^.

Level^*^	Criterion
**Optimal**	Reduces RDI<5 events per hour for at least 15min. and includes supine REM sleep without awakenings at the pressure in question.
**Good**	Reduces RDI<10 events/hour or by 50% if the baseline RDI<15 events/hour, and includes supine REM sleep without awakenings at the pressure in question.
**Adequate**	Does not reduce RDI<10 events/hour but reduces RDI by 75% of the baseline (especially in severe cases) or in cases where the criteria for optimal or good are met but no supine REM sleep occurred at the pressure in question.
**Unacceptable**	Does not meet any of the above recommendations.

Notes:

* A new titration should be considered if the good or optimum level is not reached. AASM = American Academy of Sleep Medicine; PAP = Positive airway pressure; RDI = Respiratory Disorders Index; REM = Rapid Eyes Movement.

Bilevel is the second line treatment choice for patients who do not tolerate the sensation of CPAP during titration. This method can increased tolerance than that of CPAP^98^, especially in cases requiring very high CPAP pressures (i.e., usually above 15cmH_2_O)^96^. After failure with CPAP titration, treating with Bilevel, the AASM recommends starting with the expiratory positive airway pressure (EPAP) with a pressure that eliminates OSA, and maintaining a difference in the delta between EPAP and inspiratory PAP (IPAP) of among 4 and 10cmH_2_O^96^. Of note, the use of adaptive servo-ventilation (ASV) is not yet recommended. Ongoing investigations - such as the ADVENT-HF trial - will shed the lights on the safety and effectiveness of the use of ASV for individuals with OSA and congestive heart failure (CHF)^99^.

Home titration

Home titration can be used in patients with OSA without severe comorbidities. It is performed using automatic PAP (APAP) APAP that is able to record information related to the therapy^97^. Until now, there is no standardization of conduct for home titration. The sleep PT evaluates and delimits the parameters of the initial pressure, ramp time, the pressure variation interval between minimum and maximum pressure, number of days of use and comfort measures, if necessary - but with no scientific background.

Home titration usually lasts about 7 to 14 days. Reports from the equipment used in the home titration provide detailed information on pressure behavior and residual respiratory events, as well as leakage and adherence to the therapy. These data, added to the clinical evaluation, allow the determination of the ideal parameters for the treatment^100^.

The advantages of home titration are the ease and convenience of performing the examination at home over several nights, with the management of the data on the use and effectiveness of the PAP therapy delivered through telemonitoring, when possible^98^. There are no existing guidelines in respect of the adequacy of APAP titration; but there is a set of factors that help to determine adequate automatic titration that include having an average of at least 6 hours use per day, an RDI of ≤10 events per hour, air leakage within the limits referenced by manufactures, the correction of adverse effects, and resolution of symptoms^97^.

PAP fixed pressure predictive formulas

There are formulas to predict the optimal fixed pressure of treatment. Although they are not adequately validated, they may be useful in contexts where the patient does not have access to manual or household titration^101,102^.

5.2.1.5. Educational session

Education is recommended by the AASM to increase confidence in treatment, and is an essential component in promoting adherence to PAP therapy^98^. Educational content can be provided in a number of ways that include individual consultations, telephone calls, messages, group meetings, or the provision of educational materials (pamphlets and videos).

The sleep PT should guide their patient to routine education and good sleep habits (more details in the Section 11) as they are crucial for the PAP adaptation process. Educating the patient in respect of the various data produced from their examination helps in the process of understanding the disease itself, bringing more clarity and security to the treatment. It is crucial to give the patient information on OSA in a simple way, in relation to its severity, the symptoms, the consequences of non-treatment and available treatment alternatives, including weight loss and the adoption of healthy life habits.

In the educational session, issues related to the treatment itself should be addressed, like how the PAP treatment maintains the permeability of the UA, as well as providing information on the effectiveness and safety of the treatment, always using accessible language that is easy for the patient to understand. Manage expectations and explain how PAP therapy can help to reduce drowsiness and other associated symptoms and improve mood, and quality of life, in addition to reducing the risk of comorbidities and preventing accidents.

5.2.1.6. Practice session

The practice session is the patient’s first contact with PAP therapy and comprises actions related to the choice of mask, equipment handling, training, and guidance. The sleep PT must be aware of some barriers that can directly influence the success of this first contact with therapy. PAP-induced anxiety and claustrophobia, for example, can be barriers, often requiring gradual exposure to therapy^103^.

In patients with comorbid parasomnias, like REM sleep behavioral disorder, the sleep PT must emphasize the guidelines in respect of good fixation and ease of handling the mask. In comorbid insomnia and OSA (COMISA), when insomnia is caused by fragmentation of sleep through intermittent airway obstruction, symptoms usually resolve after PAP therapy^104^. It is important to emphasize the importance of the sleep physician in guiding the treatment of OSA, especially in the presence of associated conditions such as insomnia. Specific strategies used in PAP therapy, adequate use of the ramp, may help to promote adherence to the treatment.

Choice of mask

The choice of mask is essential for good efficacy, adaptation and adherence to PAP therapy. The sleep PT should consider the route of breathing (nasal, oral or oronasal), craniofacial structural abnormalities, therapeutic pressure, as well as the preference of the patient^105^. The sleep PTs will select the most appropriate type of mask based on their evaluation of the patient and their clinical experience to ensure the most comfortable and effective treatment. Nasal route masks provide better adhesion and comfort for the patient, less side effects and residual respiratory events, as well as lower treatment pressure when compared to oronasal masks^106,107^.

In patients with hypotonia of the muscles of the face or lack of dentition, which make it difficult to close the mouth, or other reasons that hinder proper lip sealing, it may be necessary to use a chin retainer.

After choosing the mask, the sleep PT should assist in its adjustment and the positioning of the fixation strips. They should instruct the patient how to put on and remove the mask.

In the practice session, the sleep PT should set the initial pressure at a level at which the patient feels comfortable. It is strategic that the patient is told that they may feel a higher pressure when waking up during the night than experienced earlier, guiding the patient to trigger the ramp whenever this happens. After finding the initial comfortable pressure, the sleep PT should adjust the equipment to gradually increase the pressure during the mask test to the therapeutic pressure, while ensuring that there are no air leaks^108^.

Choice of equipment

The appropriate choice of equipment is influenced by several aspects arising from the results of the diagnostic examination, as well as considerations relating to comfort and whether there is a need for the use of telemonitoring resources. Each aspect should be ethically evaluated according to the situation of each patient, and considering all the information collected in the evaluation.

Information from the diagnostic sleep study

Sleep latency can be used to guide the ramp adjustment if the patient agrees that the time taken to fall sleep on the examination night reflected their routine. When the awakening index is similar to the AHI, the reduction in respiratory events produced by the treatment will solve the fragmentation of sleep.

The presence of central events in the diagnostic examination is a significant predictor of central apnea emerging during treatment^109^. The normalization of the level of oxygenation through the correction of obstructive respiratory events is one of the main therapeutic objectives in patients with OSA. In patients with an ODI much higher than their AHI or who present sustained hypoxemia in the absence of respiratory events, it is essential to perform a titration sleep study. If it is not possible, night oximetry during the PAP therapy could be used to evaluate the respiratory events.

Two pathophysiological phenotypes of OSA, supine position-related OSA and REM-related OSA, respond better to treatment with APAP. In these patients, the use of the automatic mode, where the pressure is increased only at moments of impairment of UA patency (supine and REM sleep), can be more comfortable for the patient.

The sleep PT has to understand the pathophysiology of other sleep disorders and be able to identify their presence. For example, the rate of periodic limb movement tends to increase after correction of respiratory events with PAP^110^, which can frustrate the patient’s expectation of improved sleep quality.

Comfort technologies

Comfort-related features may be required for some patients and should be evaluated individually. Some features are present only in specific equipment brands.

Expiratory relief: the goal of expiratory relief is to make exhalation more comfortable through a reduction in pressure at the beginning of exhalation^111^. To date, scientific evidence shows that there are no benefits in using expiratory relief at the beginning of treatment^98^. In clinical practice, patients who could benefit from this feature are those with greater difficulties at the beginning of treatment with PAP, especially in relation to expiration.

Heated humidifiers and breathing circuits: evidence suggests that heated humidifiers can reduce nasal resistance, decrease the level of cytokines, attenuate inflammation and fibrosis of the nasal mucosa^112^, and prevent UA dryness, in addition to significantly reducing side effects as dry mouth and UA and nasal bleeding, promoting greater comfort for the patient during the use of the device and significant improvement in quality of life^98,113,114^. The heated circuit, available in some equipment, aims to prevent condensation.

Ramp: the ramp time is the period configured from the initial comfort pressure to the optimal treatment pressure and, in most devices, can be adjusted between 5 and 45 minutes^115^. The ramp time must be configured according to the time the patient habitually takes to fall sleep. This feature can make the beginning of PAP therapy more comfortable, facilitating the onset of sleep. Some PAP equipment has a physiological ramp feature that will respond to the patients’ respiratory events and adapt to the variability of daily latency (automatic and intelligent)^116^.

Telemonitoring

Remote tracking systems have been implemented by PAP manufacturers to assess treatment adherence and effectiveness from the earliest days. Information is collected through a mobile device or modem and transferred to a database via the internet or Bluetooth connections^97^. According to the AASM, consider telemonitoring technology to direct support interventions, especially for patients who deal with difficulties in adaptation^117^.

Among the benefits of telemonitoring are immediate remote interventions that allow adjustments of equipment parameters, with a potential increase in adherence, a reduction in face-to-face visits, better quality of treatment, the correction of possible failures of use by the patient and a decrease in the rate of early abandonment of treatment^117,118^.

PAP equipment that incorporates a telemonitoring system offers the option of the use of mobile applications by patients to monitor data related to adherence, residual events and leakage, among other factors. Some of these applications provide videos and guides in order to educate the patient and improve their motivation^119^.

5.2.1.7. Adherence to PAP therapy

Adherence is the main challenge of any chronic condition control treatment. A recent study collected via telemonitoring showed that the rate of adherence was greater than 70% and the average hours of use per night was almost 6 hours in the first 3 months. In Brazil, a study reported that the rate of adherence to PAP therapy in the period of 1 year was 83%^120^.

The concept of good adherence to PAP therapy is not well defined. In the 1990s, some studies showed that the average use of PAP therapy was approximately 4.7 hours per night in adults. The 4-hour cutoff point as the minimum acceptable criterion was adopted^121,122^. Since then, although there is no formal definition, it is common to consider acceptable adherence as the use of the therapy for 4 hours per night for 70% of the nights evaluated. In any case, the criterion judged to indicate good adherence to PAP therapy will depend on the outcome being evaluated, among other factors. Several studies with other outcomes showed dose-response effects, that is, the longer the use time, the better the outcome^123-125^. Regardless of adherence cutoff points, there is evidence that any time of use is better than no use at all^126^.

5.2.1.8. Monitoring PAP therapy

The patient needs to have periodic follow-up with the aim of ensuring good adaptation, adherence and optimization of treatment. Doubts or complaints often arise at the beginning of PAP therapy, and the rapid resolution of these issues are paramount to prevent abandonment of the therapy. There is scientific evidence that long-term adherence to PAP therapy is directly influenced by the first weeks of treatment, alerting sleep PT to the importance of being as close as possible to their patients in this period^125,127^. The international guidelines on PAP therapy for the treatment of OSA recommend that patients are followed up frequently in the first months, especially in the first weeks and, after making sure that there is good initial adherence, patients should be reassessed once or twice a year by a specialized professional^98^.

The follow-up period will be comprised of periods of high, medium and low frequency follow-up to ensure the appropriate management of patients undergoing treatment for OSA with PAP therapy (Figure 3). The criteria that should be evaluated at each stage by the sleep PT are described below. In addition to these criteria, it is suggested that the sleep PT includes other possible aspects that may be relevant to each patient, taking into account their assessment and the importance of treatment personalization.

High frequency monitoring

When the patient is starting treatment, this is the time when they will have their first experience with PAP therapy at home. This period may be concomitant with automatic titration, if this is the form of evaluation adopted to establish the optimal treatment parameters. This monitoring can be performed in person or at a distance. The criteria that should be evaluated in this period to ensure the optimization of PAP therapy are described below.

Criterion 1: *Effectiveness of PAP therapy*

The evaluation of the effectiveness of PAP therapy involves the analysis of factors, such as residual respiratory events, treatment pressure behavior (when in automatic mode), leakage and time of use. It is imperative that the sleep PT evaluates in general (over several nights) and in detail (night to night), according to the capabilities of the different types of equipment. It is possible to detect whether the residual events are distributed throughout the night or at specific times, which can direct the PT to different conclusions. The overall objective is that the AHI reduces to less than 5 events/hour; yet an AHI of 10-15 can be considered acceptable in some contexts^97^. Excessive leakage can directly influence the marking of residual respiratory events by the equipment, as well as influence comfort and adherence to therapy.

Criterion 2: *Sleep habits*

The sleep PT must be aware of the patient’s sleep context, since bad sleep habits can negatively influence adherence to PAP therapy^128^. The identification of bad sleep habits can make it necessary to have a higher frequency of follow-up until the patient can follow the strategies to improve their sleep-related behavior.

Criterion 3: *Perception of sleep quality*

The patient’s perception of their sleep quality is indispensable information that may be related to adherence to therapy. The sleep PT can assess and raise awareness regarding the patient’s perception of sleep quality after treatment through simple questions or questionnaires validated for this purpose, described in Section 3.

Criterion 4: *Resolution of symptoms/main complaint*

Improvements in symptoms and complaints should be addressed and highlighted so that the patient associates these improvements with the PAP treatment. Reassessing the patient’s functioning is vital to increase their perception of the benefits of the treatment.

Criterion 5: *Management of adverse effects related to PAP therapy*

Although safe and usually well tolerated, there are some potential adverse effects of PAP therapy. Proper evaluation and monitoring can prevent the development of these events. Their recognition and a proactive clinical approach will minimize their effects on PAP adherence^129^.

Air leakage from the mask: this can occur if the mask is incorrectly attached. The sleep PT might check if the patient is putting on the mask correctly. Guidelines for positioning the head in the lateral position may be useful, as well as addressing the importance of putting on the mask with a clean face, thereby preventing oil or other substances from contributing to the leak. Changing the mask should be considered if none of these measures are effective.

Dry mouth and throat: pressurized air can promote dryness in the UA and the use of heated humidification can reduce this adverse effect^112,130,131^. Frequent opening of the mouth when using a nasal mask can cause oral dryness and a chin retainer can help to solve this issue^132,133^. If these measures are not effective, switching to an oronasal mask could be considered^132^.

Nasal congestion: nasal congestion may occur as an adverse effect of PAP therapy and a humidifier may reduce this symptom^112^.

Skin lesions: in some cases, the mask can cause skin lesions, especially on the nasal bridge. Changing the mask for another one made of a different material can help, but before doing this, check the size and adjustment of the new mask^134^, and provide the patient with information on how to sanitize and put on the mask. A replacement mask can have different pressure points, allowing previously damaged areas to recover. If these measures fail, temporary suspension of the use of the mask, or protecting the sites of the lesions until they heal should be considered.

Suffocation sensation: this can occur with some patients as soon as they put on the mask and start PAP therapy. In this case, changing the initial pressure to a level that is as comfortable as possible and adjusting the ramp time according to the sleep latency of the patient are strategies that can help. The feeling of suffocation that occurs when awakening in the middle of the night can be caused by insufficient therapeutic pressure or excessive leaks^135^. In this case, the therapeutic pressure should be reassessed, and the source of any leaks identified. The use of comfort tools, like pressure relief responsive to awakening can be an alternative measure.

Aerophagia: patients with aerophagia may present reflux, abdominal distension, flatulence, pain, and gastrointestinal discomfort^136^. Some strategies that can help are using a nasal mark instead of an oronasal mask, as well as investigating any possible gastrointestinal disorders and proper orientation regarding the time of the last meal^7,136^. Another option is to reduce the therapeutic pressure, always taking care to maintain a pressure that is optimized to reduce obstructive events and the recurrence of OSA symptoms^136^.

Criterion 6: *Patient motivation*

The sleep PT must be aware of the motivational stage of the patient in respect of the PAP therapy during follow-up, so that they can adapt their approach to ensure a lower risk of relapse.

If the patient is unable to satisfactorily meet any of these criteria, the sleep PT should reassess the situation and propose an intervention directed toward the criterion that needs to be improved. During this period, a high frequency of monitoring should be maintained. Once the patient meets these 6 criteria satisfactorily, they can then move to the next stage which consists of a lower frequency of monitoring.

Medium frequency monitoring

At this stage, it is expected that the patient will already have had a number of months of satisfactory PAP therapy, and that the frequency of contact with the sleep PT will have decreased.

Criteria 1 to 6

When reviewing the patient at this stage, the sleep PT should reevaluate the 6 criteria mentioned in the previous stage, in addition to the criterion described below.

Criterion 7: *Equipment conditions, mask, and accessories*

The mask and accessories have a certain lifespan, which can vary according to the different brands and the patient care. Some adverse effects (leakage or skin damage), can be caused by the poor condition of these materials.

The sleep PT should evaluate the conditions of the mask, the filter and other accessories, checking the need for change. They should reinforce, whenever possible, the hygiene and care guidelines for good maintenance of the equipment, the mask and the accessories.

After reassessment, if the patient does not meet any of the criteria satisfactorily, an intervention directed toward what needs to be improved should be implemented. During this period, it is recommended that a higher frequency of monitoring should take place until the resolution of the problem. The sleep PT should assess whether there is a need for new titration and/or further educational sessions. Once the patient meets the 7 criteria satisfactorily, they should be considered able to move to the next stage, which consists of a low monitoring frequency.

Low frequency monitoring

At this stage, it is expected that the patient will have undergone several months of satisfactory PAP treatment with good effectiveness and have no complaints regarding the therapy, with the equipment and accessories in good condition, and with improved symptoms and sleep habits and good motivation. It is possible to reduce the frequency of contact with the sleep PT.

Criteria 1 to 7

When reviewing the patient at this stage, the sleep PT should reevaluate all the previously mentioned criteria.

As in the previous steps, if the sleep PT identifies any criteria that are not satisfactory, a targeted intervention should be performed, increasing the frequency of monitoring until the resolution of the problem. The sleep PT should assess the need for new titration and/or educational sessions. Once the patient meets all the criteria satisfactorily, they can continue in this stage with low frequency monitoring.

The ideal follow-up model is one that meets the patient’s demands and ensures that the PAP therapy is optimized, with the maintenance of good adherence without any problems, strong motivation and improved functioning. The criteria and the frequency of monitoring presented here are the foundations that guide the good management of PAP therapy by sleep PTs. Each patient presents different personal and environmental contexts, which should be evaluated and taken into account to customize the follow-up.

### 5.3. Other treatments for OSA

#### 5.3.1 Respiratory muscle training

Some scientific basis supports the applicability of respiratory muscle training in patients with OSA. The training aims to strengthen the pharyngeal, intercostal and diaphragmatic muscles, which can reduce the collapsibility of the UA during sleep. In addition, the findings of lower functional capacity and greater fatigue of the inspiratory muscles in patients with OSA^137^ reflect muscle impairment and the need for intervention.

A systematic review and meta-analysis from 2020 showed that respiratory muscle training may be an adjunct therapy for the treatment of OSA^138^, but they highlighted the need for more studies with a higher quality of evidence and lower heterogeneity. The efficacy, indications and protocols for the application of respiratory muscle training in individuals with OSA still need to be fully established.

#### 5.3.2 Supervised programs of therapeutic exercise

Patients with OSA have worse maximal aerobic capacity and lower levels of physical activity when compared to individuals without OSA^139,140^. Longitudinal studies have reported that low cardiorespiratory fitness seems to be an important factor for the development of OSA^141^ and, low levels of exercise are associated with increased AHI^142^.

A small number of studies have been published suggesting that exercise programs can improve respiratory events during sleep, as well as being effective for improving quality of life, sleep quality and excessive sleepiness in patients with OSA. The few studies available in the literature present a wide variety of exercise protocols, but aerobic exercises, sometimes combined with strengthening exercises, seem to be effective for improving the evaluated outcomes^143,144^. No consolidated physical exercise protocol exists until now, warranting further investigations.

The sleep PT can contribute to the treatment of OSA by conducting a supervised program of therapeutic exercises as an isolated treatment strategy in less serious cases, or as a combined strategy with other therapies.

#### 5.3.3. Combined therapies

Some therapies can be combined with the use of PAP to assist in the patient adherence. Orofacial myofunctional therapy, performed by a speech therapist specialized in sleep, reinforces adherence to PAP therapy by improving the positioning and tone of the orofacial structures, in addition to helping in the sealing of the lips to prevent cases of air leak through the mouth^145,146^.

In cases where there is significant obstruction of the UA, an evaluation by an otorhinolaryngology (ear, nose and throat surgery) may be indicated to analyze the need for surgery which may favor adherence to PAP therapy^147^.

Intraoral devices, prescribed by dentists, can be used together with PAP therapy. This treatment should be considered in cases of very high therapeutic pressures that compromise adherence or produce adverse effects. Positional therapy, aims to reduce the time in a supine position during sleep, as this position is associated with an increase in obstructive events^148^.

Patients with OSA who are being treated with PAP therapy should be encouraged to participate in physical exercise programs to help to reduce symptoms and increase their quality of life, in addition to improving any comorbidities^140,149^.

The sleep PT can contribute to the treatment of OSA by conducting a supervised program of therapeutic exercises as an isolated treatment strategy in less serious cases, or as a combined strategy with other therapies.

#### 5.3.3. Combined therapies

Some therapies can be combined with the use of PAP to assist in the patient adherence. Orofacial myofunctional therapy, performed by a speech therapist specialized in sleep, reinforces adherence to PAP therapy by improving the positioning and tone of the orofacial structures, in addition to helping in the sealing of the lips to prevent cases of air leak through the mouth^145,146^.

In cases where there is significant obstruction of the UA, an evaluation by an otorhinolaryngology (ear, nose and throat surgeon) may be indicated to analyze the need for surgery which may favor adherence to PAP therapy^147^.

Intraoral devices, prescribed by dentists, can be used together with PAP therapy. This treatment should be considered in cases of very high therapeutic pressures that compromise adherence or produce adverse effects. Positional therapy, aims to reduce the time in a supine position during sleep, as this position is associated with an increase in obstructive events^148^.

Patients with OSA who are being treated with PAP therapy should be encouraged to participate in physical exercise programs to help to reduce symptoms and increase their quality of life, in addition to improving any comorbidities^140,149^.

### 5.4. Physiotherapeutic management protocol.

The suggested protocol for the management of OSA is presented in Figure 3.

### 5.5. Recommendations

Table 6 describes the recommendations related to physiotherapeutic treatment for OSA, classified according to the SORT scale^2^.

**Table 6:** Strength of recommendations taxonomy scale classifications in respect of recommended physiotherapeutic treatments for obstructive sleep apnea.

Recommendation	Strength
Motivational interventions should be performed during PAP therapy.	A
Educational interventions should be performed before and during the follow-up of PAP therapy.	A
In cases of OSA without severe comorbidities, PAP titration can be performed both in the laboratory and with APAP in the home environment.	A
In the absence of contraindications, CPAP or Auto-APAP should be used in the treatment of OSA.	A
Therapeutic exercise programs that promote improvement in AHI, EDS and quality of life and should be considered in patients with OSA.	A
The choice of the mask should be made based on a personalized assessment, advocating the use of the nasal route if possible.	B
The heated humidifier should be used in case of drying of the UA.	B
Desensitization techniques to PAP therapy should be performed in the presence of anxiety and claustrophobia.	B
Sleep PTs should indicate combined therapies that can be associated with PAP therapy for better treatment of OSA.	B
In cases of patients with an oronasal mask who present aerophagy, it should be changed to a nasal mask and the treatment pressure reviewed.	C
Telemonitoring should be used, when available, for patient follow-up in PAP therapy.	C
The ramp should be used with an initial pressure that provides the greatest comfort for patients who use PAP therapy.	C
Respiratory muscle training can be an adjunct therapy for the treatment of OSA.	There is no evidence to support the recommendation of this practice

Each patient with OSA, regardless of its severity, lives in their own personal and environmental context, has a specific level of motivation, presents different complaints and different impairments in respect of functioning. The sleep PT can help to restore the functioning of the patient through improving their sleep, after first taking into account all of these aspects and creating a personalized, facilitating environment that uses individualized strategies. The evaluation and monitoring of the patient by the sleep PT based on the model described in this Section is fundamental in the treatment of OSA and can positively influence the patient’s adherence to therapy, resulting in improved health and quality of life.

## 6. CENTRAL SLEEP APNEA IN ADULTS

Central sleep apnea (CSA) is characterized by the cessation of airflow during sleep associated with reduced or absent respiratory effort^150^. The forms of manifestation of CSA can be: 1) intermittent or isolated, occurring at different times during the night; 2) short-cycle periodic breathing, an increasing-decreasing pattern alternating with periods of central apnea or hypopnea, with a 20-40 seconds cycle duration; 3) long-cycle periodic breathing or Cheyne-Stokes breathing (CSB), an increasing-decreasing pattern alternating with periods of central apnea or hypopnea, with a 45-75 seconds cycle duration^151^. To facilitate the understanding of sleep physiology and respiratory control, as well as the pathophysiology of CSA discussed in this Section, some significant concepts are defined below (Table 7).

**Table 7:** Concepts related to the pathophysiology of central sleep apnea^152^.

	Definition
Apnea threshold	When there is a reduction of PCO_2_ during sleep to values below the apnea threshold, central apnea occurs. In general, the apnea threshold is 1 to 2mmHg below the baseline CO_2_ level in wakefulness
Arousal threshold	Respiratory stimuli can contribute to arousal from sleep during a respiratory event. Frequent arousals can cause sleep fragmentation, and sleep instability, and perpetuate unstable breathing
Loop gain	Ventilatory response to metabolic disturbance ratio Loop gain = 1 - Physiological response Loop gain > 1 - Exaggerated ventilatory response (unstable respiratory control). Loop gain < 1 - Reduced ventilatory response

Although CSA is less frequent than OSA in the general population, it is common in specific subpopulations, including patients with cardiovascular and neurological diseases and those with chronic use of opioids. These respiratory disorders rarely occur in isolation, and it is common for patients to present obstructive and central events associated with a certain clinical condition. Patients with CSA may have an obstructive component of the UA, mainly due to the suppression of respiratory flow during the central event. As there are no specific physical findings in respect of CSA, the signs associated with the presence of morbidity and the interpretation of polysomnographic findings should be strongly considered during the evaluation. The sleep PT should know how to identify the predominant patient clinical phenotype, as well as the pathophysiology involved in the development of these disorders. In the physiological process, during the transition between wakefulness and sleep (especially in lighter stages of sleep N1 and N2), when oscillations occur in ventilation control, CSA can manifest until sleep stabilizes^151,152^. CSB may be common in patients with CHF^153^, as well as ataxic respiratory patterns in patients using opioids^153,154^. In other cases, the identification of the central component is not always so evident. The presence of mixed and central events in PSG, even with a predominance of OSA, may suggest a ventilatory instability (high loop gain). Similarly, short-term respiratory events followed by arousals may be associated with a low arousal threshold^155^. The identification of these phenotypes, which can coexist and exhibit variability at night-to-night, as well as knowledge of central respiratory instability patterns and the pathophysiology of CSA are fundamental for an adequate therapeutic approach.

The ICSD-3 classifies CSA into 6 categories^20^. In this Section some of the main subtypes of CSA experienced in the clinical practice of the sleep PT will be addressed, highlighting the scientific evidence in the literature and the expertise of professionals in the field in the physiotherapeutic treatment of this sleep disorder.

### 6.1. Central sleep apnea with Cheyne-Stokes breathing

**Figure 4:**
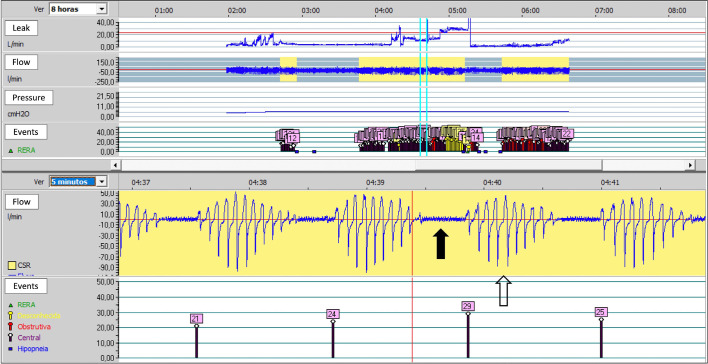
Graphical data presentation, extracted from positive pressure equipment, showing periodic pattern with Cheyne-Stokes breathing.

Notes: 5-minute sample of respiratory flow waveform, with an increasing-decreasing breathing pattern, indicative of respiratory instability. The black arrow shows a central sleep apnea, and the white arrow shows the period of hyperventilation in an increasing-decreasing format.

CSB is probably the most common CSA subtype, occurring in a large proportion of patients with CHF^156,157^ is characterized by periods of hyperventilation in an increasing-decreasing pattern, alternating with periods of central apnea or hypopnea, with the respiratory cycle duration usually exceeding 40 seconds^158,159^ (Figure 4).

In CSB, the use of continuous CPAP is effective in suppressing CSA, promoting increased stocks of O_2_ and arterial CO_2_ and a reduction in the ventilatory instability responsible for the variations in respiration characteristic of this disturbance^160,161^. In addition to increasing UA patency, the positive pressure of the CPAP is transmitted to the lungs, which may be beneficial for patients in respect of improving cardiac performance and reducing pulmonary congestion^162,163^.

ASV is a ventilatory support device designed primarily for the treatment of CSA^164^. It adjusts to the breathing phase and ensures a dynamic adaptation of the respiratory pattern^163^. ASV has presented good results regarding sleep quality and decreased daytime sleepiness, besides reducing plasma levels of the natriuretic peptide type B, compared to CPAP^165^. Although, the “Adaptive Servo-Ventilation in Patients with Heart Failure” study (SERVE-HF study) conducted in 1.325 patients with severe CHF and left ventricular ejection fraction (LVEF) <45%, randomized for usual treatment plus ASV, or usual isolated treatment, demonstrated that there was an increase in mortality due to cardiovascular causes and all-cause mortality in the group undergoing treatment with ASV^166^. Since then, the use of an ASV device has been contraindicated in individuals with reduced LVEF. For these patients, other therapeutic strategies (CPAP, oxygen therapy, and positional therapy) have been indicated.

The optimization of pharmacological therapy and cardiac resynchronization is the main therapeutic alternative for the treatment of CSA in individuals with CHF^167^, prescribed by a physician. Some studies suggested the use of respiratory stimulators (acetazolamide and theophylline) for the treatment of CSB^165,168^.

Another approach in the management of CSA with CSB is positional therapy, given the PT’s knowledge and experience in non-pharmacological and non-invasive interventions. Raising the head of the bed can reduce the rostral displacement of fluids to the lungs, contributing to a reduction in central events^169,170^.

### 6.2. Central apnea due to a medical disorder without Cheyne-Stokes breathing

Chronic clinical conditions (e.g., kidney disease, cardiovascular disease, pulmonary hypertension, stroke, and other neurological diseases) have been associated with CSA and may or may not have a pattern of CSB.

In stroke patients, obstructive and central apneas and hypopneas occur more frequently compared to the general population^171^. OSA is more common than CSA in this population, and both forms must be distinguished because the natural history and management are different. Some studies reported an increase in the incidence of CSA in the acute phase of stroke and a reduction in the frequency and severity of this respiratory disorder over time as patients’ recovery^172,173^. Studies suggested that the mechanism of CSA after stroke is a direct consequence of the lesion on central nervous system structures, which involves autonomous and volitional respiratory centers^174^.

**Figure 5:**
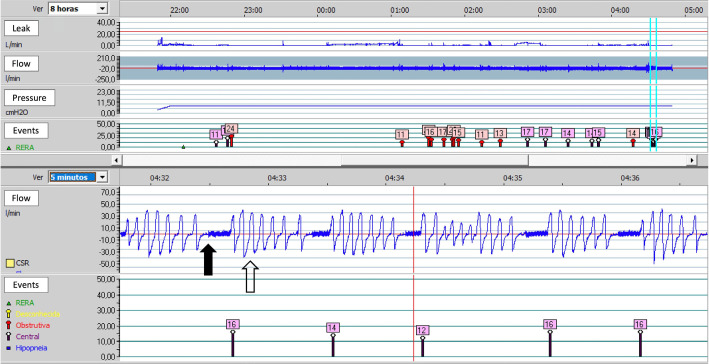
Graphical data presentation, extracted from positive pressure equipment, showing periodic pattern without Cheyne-Stokes breathing.

Notes: 5-minute sample of respiratory flow waveform of a post-stroke patient, with a periodic breathing pattern, without CSB. The black arrow shows central sleep apnea and the white arrow the hyperventilation period.

Periodic breathing with shorter cycles (without the Cheyne-Stokes pattern) occurs in individuals with stroke without left ventricular dysfunction (Figure 5).

Some observational studies have reported the positive effect of SDB treatment on stroke risk. Marin et al. demonstrated a reduction in fatal and non-fatal cardiovascular events, including stroke, in patients treated with CPAP^175,176^. Most of the evidence about early therapy with CPAP in stroke patients includes the treatment of both obstructive and central respiratory events^176,177^. Treatment of CSA after stroke, with normocapnia and ventilatory instability, includes therapy with CPAP and ASV^178,179^, although its use is still controversial in patients with predominant CSA and CHF^166^. In post-stroke patients with LVEF >45% and intolerant to CPAP, ASV can be used^180,181^. In individuals with hypercapnia and hypoventilation, the use of a Bilevel with a backup respiratory rate to ensure adequate ventilation is a treatment option^179,180^.

PAP adherence can be challenging in stroke patients, as they may have dementia, delirium, aphasia, anosognosia, pseudobulbar or bulbar paralysis, or motor impairment. Educational and behavioral strategies should be strongly employed to improve adherence to treatment with PAP, especially in the first days after stroke. Some studies described that the treatment of SDB improves stroke outcomes including in respect of severity, functional status, and recurrent vascular events^171,182,183^.

### 6.3. Central sleep apnea due to a medication or substance

Opioids are widely used to treat acute and chronic pain. When used chronically, they can cause sleep architecture changes and respiratory depression, with hypoventilation being secondary to the reduction in the ventilatory drives^184^. The use of opioids is associated with the presence of central and obstructive apnea and hypopnea, hypoventilation, periodic and ataxic respiratory patterns (Biot’s breathing), and hypoxemia^185^.

The treatment of this population of patients consists of the optimization of the drug therapy (discontinuation of the drug or dose reduction or exchange for another class of analgesic) and PAP therapy^184^. Some studies have observed that CPAP therapy was insufficient to fully control central events in this population^186^. In these cases, therapeutic alternatives (e.g., oxygen therapy, ventilatory stimulants), and other PAP modalities may be necessary^185^. In patients with CSA due to hypoventilation and non-responsive to CPAP, the use of Bilevel devices with a backup respiratory rate and ASV has indicated a better success rate. The use of Bilevel eliminated CSA in 62% and ASV in 58% of patients with chronic opioid use^187^.

### 6.4. Treatment-emergent central sleep apnea

**Figure 6:**
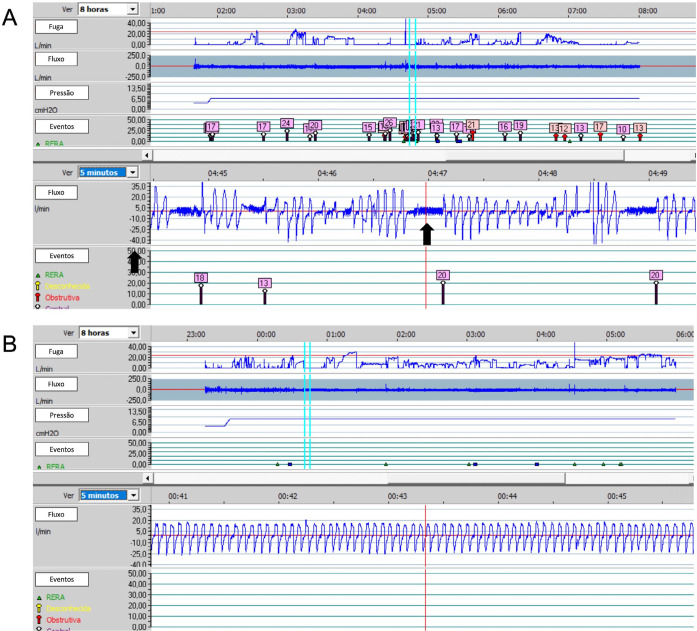
Graphical data presentation, extracted from positive pressure equipment, showing the treatment-emergent central sleep apnea.

Notes: 5-minute sample of respiratory flow waveform of a patient diagnosed with obstructive sleep apnea, showing (A) the presence of residual events of central predominance in the first days of PAP therapy, characteristic of TE-CSA; and (B) normalized respiratory pattern during sleep, after a few months of follow-up of PAP therapy. The black arrow shows central sleep apnea.

Treatment-emergent CSA (TE-CSA) previously described as “complex apnea”, refers to the development of CSA after the beginning of PAP therapy for the treatment of OSA (Figure 6A). For a diagnosis of TE-CSA, some criteria need to be followed: 1) the presence of predominantly obstructive events in the diagnostic examination; 2) the resolution of obstructive events with PAP therapy without a backup respiratory rate; 3) the emergence or persistence of central apnea and/or central hypopneas during PAP therapy with a central apnea index >5 events/hour of sleep and the number of central events at least 50% of total events; 4) The TE-CSA is not better explained by other SDB^20^.

The prevalence of TE-CSA after PAP therapy ranges between 5 and 20%^188^. Although the definition of TE-CSA involves PAP therapy, there is scientific evidence showing that TE-CSA occurs after other treatment modalities for OSA (e.g., nasal surgery^189^, UA surgeries^190^ and orthognathic surgery^191^, hypoglossal nerve stimulation^192^, and the use of mandibular advancement^193^ and tongue stabilization^194^ devices). The prevalence of TE-CSA in treatment modalities other than PAP therapy is still unclear.

The pathophysiology of the TE-CSA seems to involve an interaction between the collapse of the UA, combined with ventilatory instability and a low arousal threshold. Patients with CSA and unfavorable UA anatomy may present a narrowing of the pharynx and OSA events during CSA periods. PAP therapy resolves obstructive events and, in these cases, may reveal the underlying CSA^195^. Another pathophysiological factor of TE-CSA may be related to the PAP therapy itself. Rapid changes in the level of PAP or excessive leakage through the mask can lead to a decrease in PaCO_2_ below the apnea threshold, leading to the development of CSA^196^. A low arousal threshold can contribute to an increase in arousal during the process of adaptation to PAP therapy, resulting in greater ventilatory instability. Another mechanism is related to the effect of intermittent hypoxia, characteristic of patients with OSA. Chronic exposure to intermittent hypoxia leads to changes in the activity of peripheral chemoreceptors and is associated with a greater tendency to ventilatory instability and central events^197^.

Risk factors for TE-CSA may be related to demographic factors, comorbidities, and factors related to the PSG examination and initial treatment. In respect of personal factors, male sex, being older, and a low body mass index (BMI) is related to TE-CSA^198^. Similarly, the most common related comorbidities are CHF, coronary heart disease, and opioid use^198-200^. In respect of PSG, the factors related to the TE-CSA are a higher severity of OSA, high central or mixed apnea rates, as well as a high arousal index^198,199,201-203^. Concerning the PAP titration, the use of excessively high pressures and/or use of Bilevel, mask leakage, a high arousal index, reduced total sleep time and sleep efficiency, as well as a high residual AHI, are considered risk factors for TE-CSA^199,203^.

TE-CSA is a dynamic condition that seems to resolve after a few weeks of PAP therapy^199,204^, with a spontaneous resolution rate between 54 and 86% after a few weeks or months^203^ (Figure 6B). Approximately one-third of the patients present a persistent characteristic, and between 1 and 3% of the patients will have TE-CSA for a long time^203^. Another group of patients seems to develop late TE-CSA a few months after the beginning of treatment, with a prevalence varying between 0.7 and 4% in PAP therapy^203^. These data indicated the importance of adequate and periodic monitoring by the sleep PT, both in the short and long term.

The goal of TE-CSA treatment is to reduce the AHI and improve residual symptoms. As clinical symptoms are not included in the diagnostic criterion of ICSD-3, scientific studies investigating the treatment options for TE-CSA focus on the frequency of respiratory events as an outcome variable. Previously described, the etiology of TE-CSA is diverse, which may explain the variability of responses to treatment. The sleep PT should present an individualized approach in the follow-up of their patients with TE-CSA, considering the etiology, possible comorbidities, and symptoms, as well as the frequency of respiratory events.

During the beginning of treatment and recognition of TE-CSA, it is suggested that the sleep PT seek to maintain optimized PAP therapy and wait for spontaneous resolution of the CSA, with close and attentive monitoring (resolution between 2 and 3 months from the beginning of PAP therapy)^199,203^. Telemonitoring allows the monitoring of residual respiratory events, leak control, and adjustments of the pressure level if necessary^205^. The improvement of symptoms and a low residual AHI (<15 events/hour) favors the continuation of PAP therapy^206^. At the same time, the sleep PT must be attentive to the pharmacological optimization of the treatment of comorbidities, referring to the interdisciplinary team whenever it is deemed necessary. If TE-CSA is persistent (AHI>15 events/hour), switching to other modes of PAP therapy may be necessary, like Bilevel with a backup respiratory rate or ASV, especially in the presence of residual symptoms. Another option is oxygen therapy combined with PAP therapy, which may result in better control of TE-CSA by reducing the ventilatory drive caused by hypoxia and increasing the PCO_2_^207^. Oxygen therapy promotes a decrease in peripheral chemoreceptors activity, attenuating oscillations in ventilatory control^208^.

Acetazolamide, prescribed by a physician, seems to be associated with increased ventilation and a reduction in ventilatory instability (or high loop gain)^209^. In some cases, acetazolamide may be a treatment option combined with PAP therapy for TE-CSA, especially in cases with persistent symptoms. The effectiveness of its prolonged use has not yet been determined.

### 6.5. Physiotherapeutic objectives

- Eliminate or reduce central respiratory events during sleep;
- Identify and improve features related to poor functionality;
- Promote adherence to PAP therapy;
- Improve symptoms and the main complaint related to sleep;
- Eliminate possible adverse effects related to PAP therapy;
- Promote healthy sleep habits;
- Motivate the patient to improve their sleep.

### 6.6. Role of the physical therapist

The basis of treatment for CSA is the optimization of the underlying cause. It is crucial the interaction with the medical team for receiving supportive information about the proposed treatment. Regarding the use of positive pressure, CPAP has been recommended as an initial treatment for CSA^210^. While some authors consider CPAP therapy to be successful when an AHI<15 central events/hour of sleep are achieved, especially in patients with CHF^211^, others use a stricter criterion of AHI<5 central events per hour of sleep to indicate therapeutic success, according to the definition of CSA by the AASM^212^. Unlike the suppression of obstructive events with CPAP therapy observed in most cases, CPAP may not be able to fully eliminate central events, making the management of this disorder more challenging and requiring more parsimony and knowledge by the sleep PT about the physiology of breathing during sleep. Sometimes a higher AHI, up to 15 central events/hour of sleep, can be tolerated once the patient’s symptoms are under control and other therapeutic strategies are being used. Pressure adjustments and other ventilatory parameters should be modified with caution and care so that there is adequate time to stabilize the respiratory center in response to the therapeutic change. In addition to reducing the number of central events, the sleep PT should also assess whether there was an improvement in SpO_2_ during the use of PAP, given the cardiovascular consequences associated with intermittent hypoxia^150,213^.

Ventilatory instability associated with the high loop gain can lead to hyperventilation and hypocapnia, which, in turn, can worsen CSA, especially when CPAP is administered at higher pressures^196^, or when Bilevel devices are employed. Worsening of central events due to this phenomenon might lead to increased sleep fragmentation and low adherence to treatment.

In clinical practice, CPAP has been the first choice in the treatment of the main subtypes of CSA and can be used combined with other therapies. The AASM recommends the use of fixed or automatic CPAP for ongoing treatment of OSA in adults, but emphasizes that this suggestion was based on studies that excluded patients with morbidities, including those with CSA^98^. It is recommended the use fixed CPAP, since the automatic pressure variation can trigger periodic breathing events, especially in patients with high loop gain. The lowest possible therapeutic pressure should be used, if it is effective in suppressing existing obstructive events and, at the same time, in reducing the instability of ventilatory control and central events. Whenever possible, ventilatory stimuli should be avoided (adjustment of expiratory relief, responsive pressure relief, and automatic ramping) as they trigger instability in ventilatory control^161^.

### 6.7. Physiotherapeutic management protocol

**Figure 7:**
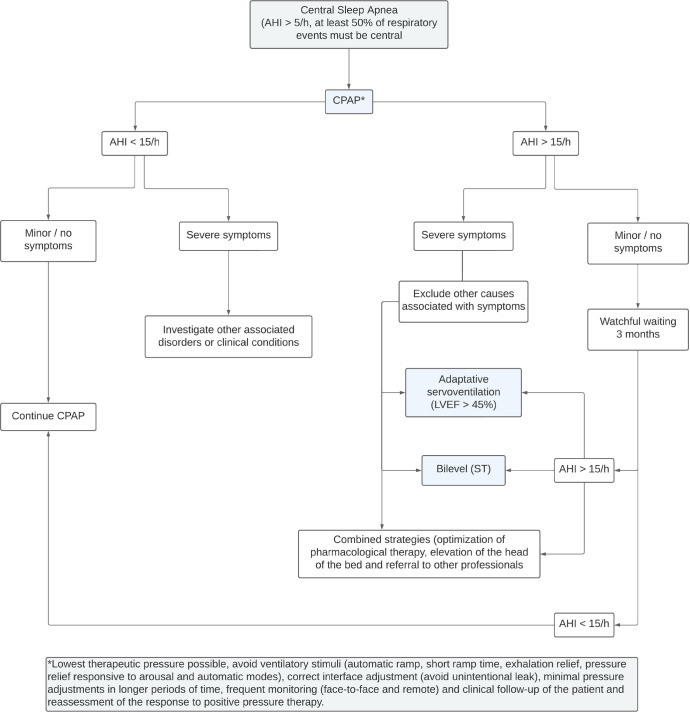
Proposed flowchart for physiotherapeutic management in the CSA treatment in adults starting with CPAP.

Notes: AHI = Apnea-hypopnea index; CPAP = Continuous positive airway pressure; LVEF = Left ventricular ejection fraction; S/T = Spontaneous-timed.

In patients where CPAP is not sufficient to fully control central events or is not tolerated by the patient, therapeutic alternatives may be necessary. The protocol suggested for the management of CSA is presented in Figure 7.

Given the context presented, the sleep PT should monitor the patient during PAP treatment, creating a bond and making them aware of the importance of the treatment and the professional partnership. In the first weeks, this close and detailed follow-up through face-to-face consultations and remote monitoring^214^, with detailed evaluation of the statistical data and the respiratory flow waveform of the equipment, ensures adherence to long-term treatment.

The analysis of the respiratory flow waveform is a tool available in some PAP equipment and provides additional detailed information about the patient’s breathing pattern. This more detailed evaluation assists the sleep PT in the identification of apnea, hypopnea, and breathing patterns distributed throughout the night, and is a necessary complement in evaluating therapeutic success. Unlike the PSG, in the analysis of the respiratory flow waveform, there is no record of respiratory effort (captured by thoracic-abdominal belts in PSG) to assist in the distinction between obstructive and central apnea. In the case of hypopneas, the AASM classifies central hypopnea by the absence of the following criteria: snoring, paradoxical thoracic-abdominal patterns, and flattening of respiratory flow during the event^212^. Most positive pressure equipment does not have the technology to identify mixed apnea. Knowing the algorithms of the main PAP equipment, as well as knowing how to interpret the respiratory flow waveform associated with the patient’s clinical history are fundamental for the identification of these respiratory events and implementing appropriate pressure adjustments. Evaluation of the presence of any airflow unintentional leakage and other specific situations that may contribute to the arousal and perpetuation of central respiratory events in patients with central respiratory instability and a low arousal threshold is warranted.

### 6.8. Recommendations

The main strategies for physiotherapeutic treatment in the management of CSA in adults are described in Table 8, classified according to the SORT scale^2^.

**Table 8:** Classification of recommendations for physiotherapeutic treatment for central sleep apnea in adults based on the SORT scale 2.

Recommendations	Strength
CPAP is indicated for the initial treatment of CSA associated with CHF.	A
Adaptive servo-ventilation should not be used for the treatment of CSA associated with CHF in adults with LVEF <45%.	A
In patients with CSA fixed CPAP should be used with the lowest possible therapeutic pressure	C
Ventilatory stimuli (automatic ramp, expiratory relief, responsive pressure relief, and automatic pressures) should be avoided whenever possible in the treatment of CSA with CPAP.	C
In patients with CSA and CSB, positional therapy with head-of-bed elevation can be used as a therapeutic alternative, combined with PAP therapy, in the reduction of central respiratory events.	C

CSA is sleep-disordered breathing associated with multiple etiologies and with various forms of manifestation. The treatment of CSA remains a challenge for most professionals in the interdisciplinary team who work in sleep. The sleep PT has an essential role, from the analysis and identification of central respiratory events in the PSG study to the proper management of PAP, with periodic monitoring and frequent reassessments, considering the patient in their entirety. To do this, knowledge of sleep and respiratory physiology, as well as the pathophysiology and clinical polysomnographic phenotypes involved in the subtypes of CSA, are fundamental for the sleep PT to be able to outline the specific goals and the therapeutic plan in the most individualized way possible.

## 7. OTHER SLEEP BREATHING DISORDERS

In addition to the SDB described in the previous Sections, there are others that can be treated the sleep PT.

### 7.1. Primary snoring

Primary snoring (PS) is defined by the ICSD-3 as a first symptom or normal variant of SDB^20^. PS occurs without episodes of apnea, hypopnea, RERA or hypoventilation. It does not cause symptoms of drowsiness or insomnia and its intensity can vary and will often disturb the sleep of bed partner^215^. Snoring adults may have a higher prevalence of cardiovascular diseases, including hypertension and stroke. Persistent snoring should not be ignored due to the physical and mental impairment it can cause when not properly diagnosed and treated^216-219^.

### 7.2. Upper airway resistance syndrome

Upper airway resistance syndrome (UARS) is an SDB characterized by increased UA resistance, associated with increased respiratory effort. UARS leads to sleep fragmentation and negative daytime repercussions (excessive sleepiness, tiredness and fatigue). The characteristic phenomenon of this condition is the awakening associated with RERA, measured by PSG coupled to an esophageal manometry sensor, or with a nasal pressure transducer cannula^220-222^.

### 7.3. Hypoventilation syndromes

Hypoventilation is defined when there is an increase of PaCO_2_>45mmHg. In obese patients, among the respiratory repercussions, obesity hypoventilation syndrome (OHS) is noteworthy^223,224^.

#### 7.3.1. Overlap syndrome

Overlap syndrome describe the coexistence of OSA and chronic obstructive pulmonary disease (COPD). The clinical picture of OS is different from standard OSA, especially in respect of the presence of more severe sleep-related hypoxemia^225^. The combined effects of COPD and OSA result in a worse prognosis compared to patients with only one of these diseases. During sleep, they present more frequent episodes of oxygen desaturation and greater total sleep time with hypoxemia and hypercapnia^226^. Apnea events are associated with deeper hypoxemia and a greater chance of developing cardiac arrhythmias, pulmonary hypertension, and heart failure. These complications develop earlier in patients with COPD and OSA than in those without OSA^227^. The deteriorating lung function associated with an increased AHI worsens the outcomes in patients with overlap syndrome, who also have higher mortality and hospitalization rates compared to individuals with only COPD^227^.

#### 7.3.2. Hypoventilation associated with neuromuscular disease

Neuromuscular diseases (NMDs) may cause respiratory muscle weakness leading to hypoventilation. Muscular weakness of the diaphragm and the accessory muscles causes reduced tidal volumes, resulting in hypercapnia. There is a reduction in cough capacity and management of pulmonary secretion that is associated with expiratory muscle weakness, which can lead to hypoventilation. During sleep, hypoventilation is intensified when in the dorsal decubitus and during REM sleep stage, which impair the movement of the diaphragm and produce muscular atonia, respectively^228^.

### 7.4. Physiotherapeutic objectives

Reverse the main physiological abnormalities that lead to disturbances and optimize gas exchange;

- Guide, monitor and conduct treatment through behavioral guidance;
- Improve the quality of life of the patient and their bed partner, relieve symptoms, reduce morbidity, and decrease mortality;
- Promote proper ventilation during sleep;
- Improve aspects of functionality.

### 7.5. Role of the physical therapist

#### 7.5.1. PS and UARS

The main treatments available to the sleep PT in respect of these conditions are CPAP and behavioral measures, which include:

i) Positional therapy: indicated for patients with worsening of the condition when sleeping in supine position. Sleeping in the lateral decubitus (right and left) and/or with elevation of the head of the bed by in 30º is recommended. Positional therapy is considered a simple and inexpensive technique and can be used alone or as an adjunct treatment with other methods^229-231^;

ii) Weight reduction: in obese patients, weight reduction is an significant treatment and the standard recommendation^222^;

iii) Reduction in alcohol consumption, use of sedatives and smoking: alcoholic beverages and sedative medications increase the awakening threshold and promote longer obstructive events. These substances have muscle relaxant potential, increasing the collapse of the UA and, consequently, worsening the preexisting condition. The patient should be advised to stop smoking as it can cause edema, which increases airflow resistance, reduces airway caliber, and promotes dysfunction of the UA^232-234^;

iv) Sleep hygiene: the relevant guidelines are described in more details in the Section Section 11.

The use of CPAP is indicated in the treatment of PS and UARS. It is usually reserved for patients whose condition does not improve following the use of behavioral measures or an intraoral device^235^, prescribed by a dentist. CPAP has been the primary therapy prescribed for UARS. However, its effectiveness is still limited due to low patient adherence and there is a lack of randomized controlled trials evaluating this type of treatment^235-237^. Existing studies showed that CPAP significantly reduces the symptoms of snoring, nocturnal awakenings, drowsiness, and fatigue. Studies using PSG have indicated a reduction in sleep fragmentation, sleep onset latency and increased NREM N3 stage sleep^237^. The CPAP treatment pressure used should be the lowest to eliminate the flow limitation while keeping the UA open. The mean therapeutic pressure found was 7cmH_2_O, with a range of 4-9cmH_2_O^220^, and greater adherence to automatic CPAP was found compared to fixed CPAP^238^, both modalities presenting with a reduction in daytime sleeping. The CPAP pressure should be the lowest to eliminate the flow limitation and maintain UA open^220,237^.

#### 7.5.2. Obesity hypoventilation syndrome

This condition can be treated with CPAP or non-invasive ventilation (NIV) with 2 levels of PAP (Bilevel), with or without respiratory rates^239^. Initial treatment with CPAP is recommended for OHS if clinically stable, and if PaCO_2_ is unchanged. If either of these conditions are not met, the Bilevel treatment should be used^240^. The efficacy of treatment was demonstrated by a reduction in respiratory events and the maintenance of O_2_ saturation>90% associated with clinical improvement. However, regular blood gas analysis showing a PaCO_2_<45mmHg, and a pH>7.35 are significant^241^.

In patients who are refractory to treatment with CPAP or who do not have OSA, the use of Bilevel treatment is recommended. The pressures must be adjusted individually, but the highest efficiency is usually achieved with delta values of pressure >8cmH_2_O^242^.

To date, there is no evidence that proves the superiority of the use of one mode of positive pressure over the other. Both CPAP and Bilevel appear to be equally effective in improving diurnal hypercapnia in patients with OHS without severe nocturnal hypoxemia. When compared, both have been shown to improve the PaCO_2_, sodium bicarbonate levels, clinical symptoms, and PSG parameters^233,239,243^. However, patients using Bilevel had better subjective sleep quality and presented a slight improvement in psychomotor performance than the patients using CPAP^243^. Treatment with CPAP reduces nocturnal changes in blood pressure in patients with OHS with underlying OSA, and this improvement has been shown to be greater in patients with greater adherence to CPAP^244^.

Oxygen therapy should be used if O_2_ saturation levels <90% persist. Given the possibility of the hypoventilation having a central cause, the Bilevel treatment can be adjusted to ensure minimal respiratory frequency and volume^242^.

#### 7.5.3. Overlap syndrome

The use of PSG for the titration of PAP pressure is indicated when starting the treatment of patients with overlap syndrome to control the abolition of respiratory events and verification of the correction of hypoxemia, prescribed by a physician. Treatment of overlap syndrome should be started with CPAP^241^. Among the benefits of CPAP in this profile of patients are the correction of AHI, improvements in nocturnal hypoxemia and hypercapnia, a reduction in excessive sleepiness, and improved pulmonary mechanics demonstrated by a reduction in respiratory work by minimizing insufflation^245^. The use of CPAP was effective in improving lung function in respect of forced expiratory volume in 1 second (FEV_1_) and forced vital capacity (FVC)^246,247^. When the hypercapnia is sustained in a patient who is not responsive or intolerant to treatment with CPAP, the use of Bilevel should be considered. The use of supplemental oxygen may be necessary concomitantly with the use of CPAP or Bilevel for patients with overlap syndrome with a more severe COPD and *cor pulmonale*, and who already have sustained diurnal hypoxemia. Regardless of the method used, adherence to the PAP therapy is directly related to the success of the treatment.

#### 7.5.4. Hypoventilation associated with neuromuscular disease

Most of the literature addresses NIV for the treatment of amyotrophic lateral sclerosis but also includes other NMDs. Studies indicated that NIV improved gas exchange, subjective sleep quality, the ability to perform activities of daily living and increased quality of life^248,249^. It is recommended that NIV is used in the presence of symptoms (excessive sleepiness, headache, fragmented sleep, among others), associated with the progression of the disease, and decreased muscle strength^250^.

In patients with NMDs, the ventilatory therapy of choice is NIV, which may be in Bilevel mode, with or without *back-up* frequency, or supported pressure with guaranteed average volume. When ventilating the patient, the aim should be to reduce respiratory overload, increase volume minute and reduce hypercapnia. In patients requiring respiratory frequency, there is no indication of CPAP or Bilevel without back-up frequency^251^. Oxygen therapy associated with NIV, rather than oxygen therapy alone, is recommended, as it decreases PaCO_2_ during the day, and increases the PaO_2_, the maximum inspiratory pressure, vital capacity and survival^249^.

### 7.6. Behavioral measures

Guidance on behavioral measures should be the first step taken to meet the needs of the patient, although evidence suggests that the effectiveness of these measures can be low (medium and long-term), partly due to poor adherence to the guidelines.

### 7.7. Recommendations

The recommendations are based on the SORT scale ^2^ (Table 9).

**Table 9:** Classification of the recommendations for the use of physiotherapeutic treatment for primary snoring, upper airway resistance syndrome and hypoventilation syndromes.

Recommendations	Strength
NIV is recommended for patients with NMDs.	A
In patients with overlap syndrome, treatment can be started with CPAP. For those patients who do not respond to CPAP or present hypercapnia refractory to CPAP, the use of Bilevel is recommended.	B
CPAP should be used in patients with PS and patients with UARS.	C
Positional therapy should be used to minimize PS.	C
In patients with OHS treatment can be started with CPAP, if clinically stable.	C
In patients with nonstable OHS or patient’s refractory to treatment with CPAP or without OSA, Bilevel should be used.	C

The treatment of PS, UARS and RERA is still a challenge for the PT, as there is a lack of evidence of the most effective therapies. In OHS, NMDs and overlap syndrome the efficacy of PAP treatment is well established. In hypoventilation syndromes, PAP is recommended if patient is in stable conditions. In NMDs, a NIV is the treatment of choice.

## 8. SLEEP BREATHING DISORDERS IN PEDIATRICS

Pediatric SDB comprises a variety of disorders including: PS, UARS, hypoventilation, and OSA, which is the most severe type of SDB^252,253^. The first-line treatment of SDB in pediatric population is adenotonsillectomy, with the use of PAP being the second option.

### 8.1. The most common pediatric sleep breathing disorders

PS is characterized by soft palate vibratory noises during the inspiratory phase: it results from the partial obstruction of the UA and is often associated with OSA or UARS. The main causes of PS are adenotonsillar hypertrophy, obesity, respiratory nasal obstructions, and upper respiratory tract infections. Snoring may result in oral breathing, dry mouth and lips, and difficulty swallowing, halitosis and dyslalia^252^.

UARS presents increased respiratory muscle effort during sleep due to excessive UA resistance and negative esophageal pressure^254^. Breathing efforts are associated with awakening and fragmentation of sleep. The clinical consequences include poor weight development, reduced school performance, diurnal irritability, and poor development in height due to the reduced secretion of growth hormone. These clinical signs can indicate the presence of UARS, which can be confirmed by PSG^252^.

Obstructive hypoventilation is defined as prolonged hypoventilation associated with hypoxia and hypercapnia, without complete airway obstruction^255^. In children, there is a different pattern in respect of the recruitment of dilator muscles, which is characterized by greater muscle activation capable of preventing complete airway collapse. Another significant difference between prolonged hypoventilation and true OSA is that it has less effect on the structure of sleep, with children affected by obstructive hypoventilation rarely presenting awakenings^252^.

OSA in the pediatric population is characterized by prolonged partial obstruction of the UA, typical of obstructive hypoventilation, interrupted by total obstruction with hypoxemia^252^.

The etiopathogenesis of SDB in children may be anatomical and neurofunctional (nasal obstruction, neuromuscular variations, soft tissue impairment and reduced skeletal growth), and obesity may also be a cause^256^.

### 8.2. Pediatric sleep breathing disorders treatments

A team approach is essential, and should all be involved in the treatment of pediatric SDB (adenotonsillectomy^257^ and intranasal corticosteroids prescribed by physician (e.g., in patients being treated with PAP therapy, in cases of nasal obstruction, intranasal corticosteroids, which promotes adherence to treatment^258^), orthodontic treatment, performed by dentists, can be used as an alternative to PAP therapy^259^, and myofunctional therapy, performed by speech therapists^260^. The physiotherapeutic approach is PAP therapy, recommended as a treatment for children if the patient is ineligible for adenotonsillectomy surgery, or if OSA persists after surgery. PAP therapy may also be indicated for patients with SDB and other associated diseases, (i.e., obesity, Down syndrome, and craniofacial abnormalities). In general, a single surgical procedure is preferable to lifelong treatment with PAP, to which adherence in general can be low because of the discomfort, and this is even more problematic in children who tend to be less cooperative in respect of treatment^261^.

#### 8.2.1. Special conditions

Children with syndromes, neuropathies or craniofacial anatomical changes may be susceptible to SDB, with their etiopathogenesis and treatments being individualized according to each situation. Children with changes in neurological development are at risk of developing SDB^262^.

##### 8.2.1.1. Down syndrome

Children with Down syndrome have a combination of factors that predisposes OSA, like reduced airway diameter, enlarged tonsils and adenoids, macroglossia and hypotonia, in addition to a tendency to be overweight. The surgical treatment of OSA is complex because airway obstruction occurs in several places, and more than 50% of patients will have residual OSA after surgery^263^. PAP therapy is the first choice for this population, although it is often not well tolerated. The use of PAP therapies and/or other therapies, high flow nasal cannula and hypoglossal nerve stimulation may be alternative treatments in cases with problematic adherence to PAP^264^.

##### 8.2.1.2. Cerebral palsy

Children with cerebral palsy have a higher risk of SDB, especially OSA because of abnormal muscle tone of the UA, a disproportionate anatomy of the middle third of the face or mandibular changes, a primary central abnormality affecting central control of breathing, obesity or drugs that depress the UA maintenance muscles. Children with cerebral palsy also have difficulty sleeping. Endogenous dysfunction in the hormonal release necessary for the maintenance of circadian rhythm (i.e., abnormal melatonin secretion), and electroencephalogram results have shown decreased REM sleep stage and abnormal sleep spindles the presence of comorbidities in children with cerebral palsy such as epilepsy, intellectual or sensory impairment (vision or hearing) and the use of medications impacting sleep^265^. Moreover, the presence of pain may contribute to sleep problems, especially in children with severe changes in muscle tone^262^. Treatment needs to be individualized and depends on neurological abnormalities and the site of obstruction. PAP therapy may be considered, and other management discussed with the sleep health team, such as adenotonsillectomy, drugs to control muscle tone, as well as the management of hypersalivation, control of obesity and gastroesophageal reflux.

##### 8.2.1.3. Neuromuscular diseases (NMDs)

The involvement of the respiratory system in patients with NMDs is the most serious complication. Nocturnal hypoxemia is observed in most patients. NMDs patients are especially vulnerable to SDB due to several factors: the inability to inhale deeply which leads to microatelectasis, increased right-left shunt, decreased lung compliance and increased respiratory load; muscle atrophy, resulting in a decreased range of motion and intra-articular adhesions that may lead to a stiff rib cage; thoracic scoliosis which may impair respiratory mechanics; and weakness of the respiratory muscles that can increase the workload of the diaphragm, causing hypoventilation and increased PaCO_2_^266^.

Patients with nocturnal hypoventilation complain of restlessness, no recovering sleep, vivid dreams, lack of concentration, daytime sleepiness, and mood disorders. Hypercapnia can cause headache, drowsiness, confusion, and lack of appetite^266^. The monitoring of CO_2_ in patients with NMD is critical. Oximetry can be performed when capnography is not available^267^. PTs can measure maximal inspiratory pressure using manovacuometry, with a pressure ≤41cmH_2_O being associated with hypoventilation, and ≤36cmH_2_O correlated with respiratory failure^268^.

Hypoventilation is the most common event in patients with NMDs during sleep. The events represent hypopneas that are not obstructive and central, because the electromyographic activity of the entire musculature of the body is present but is reduced by the impairment of the motor unit^269^. Hypoventilation first appears during the REM sleep stage and with the progression of the disease can be observed also in NREM sleep stages. Although there is not a standard classification of hypoventilation in the literature, hypoventilation is considered as^267^: i) PaCO_2_ or PetCO_2_ (partial end-tidal carbon dioxide) or PtcCO_2_ (transcutaneous CO_2_ pressure) >55mmHg, for >10min; ii) an increase in PaCO_2_ or PetCO_2_ or PtcCO_2_ >10mmHg to a value >50mmHg, for >10min.

In addition to hypoventilation, OSA is common in patients with NMDs due to macroglossia, bulbar dysfunction and weakness of the pharyngeal musculature^267^. The main treatment of SDB in patients with NMD is PAP. Preventative nocturnal PAP therapy can be introduced even before the appearance of hypercapnia and can delay the development of hypercapnia by 4-5 years^270^. Bilevel using a nasal mask can be used to successfully treat SDB associated with neuromuscular diseases^271^. A recent systematic review and meta-analysis found that sleep parameters improve with the long-term use of Bilevel in this population^272^.

##### 8.2.1.4. Congenital craniofacial malformation

Children with craniofacial abnormalities are at increased risk of OSA due to anatomical changes, and some may also have CSA. The mechanisms of CSA are not well understood, but there is a hypothesis that it is the result of increased intracranial pressure in the respiratory center, anatomical changes or OSA itself, which may increase intracranial pressure by increasing CO_2_, which is a potent vasodilator^273^. Malformations, such as cleft palate, micrognathia, craniosynostosis, and hypoplasia of the middle third of the face may occur alone or as part of a syndrome. OSA in children with major impairments, including cognitive delay and poor suck-swallow-breath coordination, was related to a worse quality of life^274^, and should be treated by multiprofessional team^275^. This population is complex due to its heterogeneity, so individual conditions should always be considered in respect of the treatment^261^.

Some treatments are like those used in children with OSA but without NMD, such as adenotonsillectomy and use of PAP therapy. There are some specific procedures^274^ the insertion of a nasopharyngeal airway (a tube from the nostril to the oropharynx), which acts as a stent of the UA and physically prevents collapse in this region; this option of a tracheostomy^276^ should be considered in more severe cases and discussed with the physician.

For some children, for example those with Pierre Robin or micrognathia, sleeping in different positions, such as in a prone position or lateral decubitus, can reduce obstruction at the tongue; the use of positioning as a therapeutic intervention has been pointed to be successful in 25% to 66% of cases^277^. Adenotonsillectomy is often not curative in patients with craniofacial changes, and the use of PAP therapy can be difficult to implement in these children, the combination of procedures can be an alternative.

The use of PAP therapy can be challenging in children with craniofacial changes for several reasons. In infants, the available interfaces are few, and this is even more difficult for those with craniofacial syndromes and nasal deformities. Those who have undergone facial surgery may have increased sensitivity. However, PAP therapy has been described as successful in children with micrognathia and OSA secondary to palatoplasty^274^.

### 8.3. Physiotherapeutic objectives

- To provide guidance to parents and patients on treatment, equipment, and accessories;
- To achieve a good adaptation of the patient to therapy by aiding in the choice of equipment and accessories and continuous monitoring;
- To increase adherence to treatment by making necessary adjustments over time, following the evolution of the child and the underlying disease.

### 8.4. Role of the physical therapist

Once it has been decided that PAP therapy is the treatment for the child, it is recommended that several steps should be taken in respect of the preparation for its use, such as the evaluation of the presence of chronic nasal obstruction, education of the parents/caregivers and the child, as well as desensitization strategies.

The role of the PT is to perform a clinical evaluation, including the use of Altmann’s millimeter mirror to identify any possible signs of a nasal obstruction, and, if identified, to refer the patient for evaluation by an otorhinolaryngologist.

The education of parents or caregivers, and of those children and adolescents to understand the goals of the treatment, are vital to ensure the success of the treatment. The children of engaged parents, who understand the benefits of treatment, have greater adherence and better results. In specific cases, psychological follow-up may also be indicated^278^.

Desensitization strategies that help the child to gradually become used to the treatment are indispensable. Strategies involving a multidisciplinary team have been shown to be more effective in increasing tolerance to the use of PAP^279^. A variety of training techniques that include positive reinforcement, gradual exposure to the use of PAP and the use of the equipment in association with pleasurable activities for the child are described in the literature. The current literature does not suggest that any specific desensitization strategy is superior to any other^279^. Regardless of the method, desensitization strategies should be playful in nature. The use of playful elements that the child likes, such as characters, the use of masks with colored covers, and stories can help the child to accept the treatment. Initially, the equipment should be demonstrated, and before being placed on the face, the child should be exposed to the experience of pressure, for example, on the hands. After the child becomes used to the equipment, the use of the mask in the region of the face should be explored, initially without it being fixed, only being attached properly when there is good acceptance from the child. The PT should be aware that this process may take longer for certain children and may take more than 1 session of desensitization.

#### 8.4.1. Choice of mask and equipment

Choosing the right mask is essential to minimize discomfort and leakage and thus improve adherence to treatment and its effectiveness. Several models are available on the market, including oronasal, nasal, and “pillow” type masks, in different formats. In general, nasal masks are best tolerated, since they allow the child to talk, as well as to be able to cough during use. The oronasal mask, in addition to being less comfortable, may predispose the child to episodes of bronchoaspiration in cases of hypersecretion, and to episodes of vomiting^7^.

When choosing the type of mask, consider the risk of changes in craniofacial growth over the long-term, especially hypoplasia of the middle third of the face, caused by the pressure of the mask. It is essential that patients alternate the use of different types of masks when PAP is used over a long period as each type of mask produces pressure at different points on the face.

In respect of the type of positive pressure modalities used, there are 2 alternatives: CPAP and Bilevel. In the pediatric population, fixed pressure equipment is commonly used, as it offers greater pressure stability. In respect of the pressure to be used, it is suggested that this be titrated in a sleep laboratory. There is lack of studies regarding the use of auto-CPAP in children to determine whether automatic equipment is safe and effective in this population^279^. The use of Bilevel increases the chances of good adherence to treatment^280^. In practice, 2 levels of pressure are usually indicated when high pressures are required to abolish respiratory events, or when the patient presents hypoventilation, as in those with NMDs.

#### 8.4.2. Monitoring of results

Monitoring the results of PAP treatment is essential so that the therapy can be adjusted to maximize its effectiveness, and to ensure good adherence. Among the data that deserve the greatest attention, residual AHI analysis (which ideally should be less than 1 event/hour), data related to leaks, and to the duration of use, are the most important items^261^.

Several recommendations made in a study of children with NMDs^266^, can be considered equally applicable for all users of PAP therapy: i) avoid leaks as much as possible, as leaks can lead to asynchronies such as auto-triggering and prolonged insufflation and reduced sleep quality; ii) asynchronies may increase wakefulness and desaturation, impair sleep architecture, and reduce adherence to treatment; and iii) ineffective effort, usually associated with higher levels of blood pressure support and respiratory rate, can cause dynamic hyperinflation.

The use of PAP therapy in children involves structures that are growing and developing, consequently, changes in respiratory events and pressure levels may be required. Close monitoring of the treatment is essential to ensure that adequate adjustments are made.

### 8.5. Physiotherapeutic management protocol

**Figure 8:**
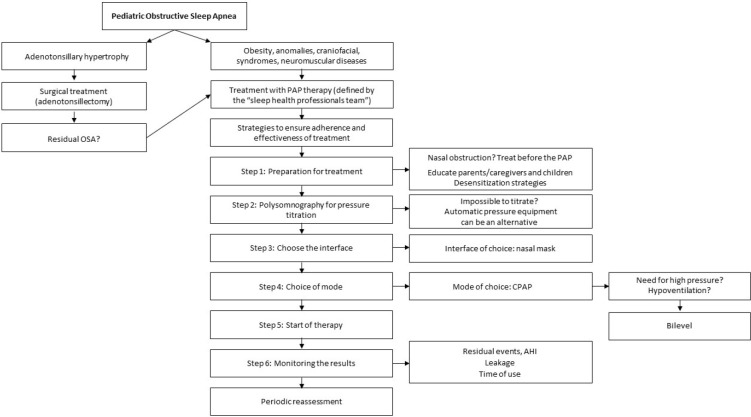
Suggested protocol for PT evaluation.

There is no consensus in the literature as to the precise periodicity of evaluations but there is agreement that they should be carried out periodically and that the periodicity should be individualized for each case (Figure 8).

### 8.6. Recommendations

Once PAP therapy is selected as the treatment of choice, the PT is critical to ensure adherence so that the goals are achieved. In children, the definition of adherence to treatment through PAP therapy is variable and generally considered the same as in adults, that is, ≥4 hours/night, in 70% of the nights, over a period of 30 days^278^.

Recommendations for improving adherence to the treatment and its effectiveness in children can be broken down into recommendations related to preparation for treatment, the choice of interface and equipment, and monitoring of results, classified according to the SORT scale^2^ (Table 10).

**Table 10:** Classification of physiotherapeutic strategies for the treatment of sleep breathing disorders in pediatrics based on the SORT scale.

Recommendation	Strength
Objective monitoring of results (residual AHI, leakage, time of use).	A
Bilevel: recommended in cases of hypoventilation.	A
Mode of choice: PAP, fixed or automatic pressure equipment.	B
Guidance on the treatment of nasal obstruction when present.	B
Education of parents/caregivers and the child.	B
Desensitization strategies.	B
Nasal route for PAP.	There is no evidence to support the recommendation of these practices.
Alternating use of masks with different pressure points.	
Subjective monitoring of results (application of questionnaires, analysis of the opinion of parents when the improvement of signs and symptoms).	

Although PAP therapy is not the first choice for most children with OSA, when indicated, it should be closely monitored to ensure good adherence to the treatment. In this population, encouraging adherence to the treatment presents difficulties, and strategies that use playing and storytelling as their main aspects appear to be the most effective and should be prioritized. Special attention should be paid to the relationship between masks and changes in craniofacial growth.

## 9. SLEEP BRUXISM

Sleep bruxism (SB) is a parafunction oromotor habit characterized by grinding of teeth and rhythmic masticatory muscle activity^20,281,282^. It is considered a central (pathophysiological and psychological), peripheric (morphologic) and proprioceptive dysfunction^283^. SB can produce excessive mechanical stress, which is a critical risk factor for dental fracture, periodontal disease, and articulatory disorders and can lead to chronic pain and limitation of mandibular range of motion. Depending on its duration and intensity, it can affect the temporomandibular joint (TMJ), the cervical spine, and the face and neck muscles. There is evidence of co-contraction of the TMJ and cervical muscles during SB, indicating a functional relationship between the muscles, or between muscle chains^284^.

The “multi P” approach is the most commonly used for the management of SB, and employs the “5 P’s”, namely: oral appliances (i.e., plates), counseling/behavioral strategies (i.e., psychology/pep talks), centrally acting drugs (i.e., pills), team collaboration (i.e., professional), and physical therapy (i.e., physiotherapy)^285^. We emphasize the need for diagnosis made by a sleep dentist prior to PT treatment, regardless of the presence of TMJ dysfunction (TMD) or degenerative TMJ diseases, which are considered different entities. The “multi P” approach requires collaboration between professionals, and uses strategies that include: i) central approaches, such as the use of medications^286^, and cognitive behavioral therapies^287,288^, advice on sleep hygiene^288^, habit reversal techniques^289^, and biofeedback^290^; and ii) peripheral approaches, such as the use of occlusal splint and mandibular advancement devices^291,292^, as well as botulinum toxin^293^. The physiotherapeutic approach can act as an adjunct to other therapies or be used alone depending on the case. PTs can identify and treat the symptoms of SB that are shown in gray in Table 11.

**Table 11:** Symptoms indicative of sleep bruxism. In blue, symptoms that can be treated by physiotherapy.

Squeaking/clenching noise during sleep.
Teeth clenched when waking up.
Feeling of tension or stiffness in the muscles of the face.
Waking up with restricted movement/discomfort in the chewing and TMJ muscles.
Difficulty in opening the mouth.
Complaints of TMJ and cervical pain.
Morning tension headaches.
Hypertrophy of the masseter and temporal muscles.
Changes in facial symmetry.
Excessive tooth wear, including loosening or fracturing of teeth.
Injury to the edge of the tongue and the inside of the cheeks.
Sensitivity in the teeth and or gums upon waking.

Note: TMJ = Temporomandibular joint.

### 9.1. Physiotherapeutic objectives

- Provide adequate management of pain (whether acute or chronic);
- Reduce muscle activity;
- Rehabilitate the function of the TMJ (increased range of motion, mobility and mandibular strength of the musculature involved, including muscles antagonistic and synergistic to the movements of the TMJ);
- Normalize the range of motion and positioning of the TMJ or associated joints due to postural disorders (cervical and thoracic spine, shoulders, reported pain pattern and presence of trigger points in other regions);
- Relieve mechanical stress;
- Normalize proprioception and muscle balance;
- Improve parafunctional and oral habits, and prevent muscle compensations;
- Maintain morpho- and physiological function of joints;
- Avoid the involvement of other regions (such as the axial region) and chronic symptoms;
- Improve/educate in relation to proprioception in the masticatory muscles.

### 9.2. Role of the physical therapist

Table 12 summarizes evidence on the treatment of SB.

**Table 12:** Physiotherapeutic treatment for sleep bruxism symptoms.

Physiotherapeutic modality		Parameters	Outcomes
**Manual therapies**	Awareness through movement (habit reversal technique, Feldenkrais method)^294^	26 children intervention vs. control 3h session 10 sessions 1x week	Improvement of the cervical anteriorization Improved control of head movement ↓Pain ↑ROM mandibular
	Manual therapy for masticatory muscles (intra and extraoral) + cervical spine *vs.* manual therapy + KT at masseter muscle^295^	38 adults intervention *vs.* control 30mins. session 1 session	↓Pain threshold at pressure on masseter and temporal muscles ↓Thickness and stiffness of masseter and temporal muscles ~ Perception of pain
	KT *vs.* occlusal splint^296^	34 adults Sessions at the end of the day, every day 5 weeks 35 applications withdrawn in the morning	↓Muscle pain ↓Perception of pain (VAS) ↑ROM mandibular KT is as effective as occlusal plate ↑Pain threshold of masseter and temporal muscles
	KT *vs.* manual trigger-point release^297^	60 patients	Instant analgesic effect after application of KT ↓Intensity of pain
	KT^298^	1 application	↓Intensity of pain ↓Muscle activity 24h and 48h after application
	Dry needling at latent trigger points (masseter and temporal)^299^	16 adults 1 session Follow-up of 1 week	↓Intensity of pain ↓Threshold of pressure to pain ↑ROM mandibular immediately post session and after 1 week
	PNF + myofascial maneuvers *+* home exercises *vs.* myofascial maneuvers *+* home exercises *vs.* occlusal splint^300^	52 patients Does not mention the length of sessions or the number of sessions 2 weeks/6 weeks + 3 sets of 10 home repetitions	The combination of PNF therapies + myofascial maneuvers + home exercises: ↓Pain in the TMJ Improved oral habits ↑Mandibular ROM and mobility
	Mobilization and manipulation of high cervical vertebrae^301^	1 child 2 sessions	↓Headache ↓Cervical pain ↓Report of bruxism (parents)
**Therapeutic exercises**	Use of Pro-fono device + encouragement, education, stretching, muscle relaxation + home exercises^302^	39 adults intervention vs. control 1-3 series of 15-20 repetitions of isometric contraction	↓Muscle activity of the masseter muscle activity of the buccinator (intercalated contraction) ↓Intensity of pain ↑Function
	Static stretching of masticatory muscle^303^	24 adults (SB without pain) intervention *vs.* control 10 days	↑ROM mandibular ↑Threshold of pain at pressure ↑ # SB episodes/h of sleep Ineffective therapy to reduce SB in the absence of pain and dysfunction
**Electrotherapy**	TENS *vs.* occlusal splint^304^	24 adults TENS: 45-60min. session every 2 days/15 sessions Occlusal splint: 45 days, 24h/day	Therapies did not improve SB symptoms
	Auricular TENS in specific auricular areas associated with the vagus nerve^305^	10 adults 4 sessions	↓SB tonic index (PSG) ↓Contraction time of SB episodes (PSG) ↑Vagal tone after each stimulation session (HRV)
	TENS (electrode in the masseter; F: 50Hz, τ: 0.5 msec, intensity: maximum tolerable by the patient) *vs.* Micro currents (electrode in the masseter; F: 0.5Hz, intensity: maximum tolerable by patient)^306^	60 adults TENS *vs.* microcurrents 20min. session 7 sessions	Results favor microcurrent therapy: ↓Pain Improved sensitivity
	TENS *vs.* relaxation training and muscle awareness^307^	23 adults 20-30min. session 2/week 20 sessions	Results favor relaxation training therapy and muscle awareness: ↓Pterygoid-masseter activity (EMG) ↓# breaths per minute ↑ROM mandibular
	Different electrical biofeedback modalities^308^	Meta-analysis Inconsistency between parameters, except significance in surface electrical stimulation	↓SB episodes with electrical stimulation verified by (EMG) after 5 nights of use
**Acupuncture**	Acupuncture^309^	4 adults 1 session monitoring for 3 days	↓Pain (VAS) ↓EMG activity (masseter, ascending trapezius) Improvement of the muscular symmetry of the temporal muscle Improvements remained for 72h
	Acupuncture with and without occlusal splint^310^	29 adults (SB and pain) acupuncture *vs.* acupuncture + occlusal splint	Acupuncture only: ↓Pain
**Photobiomodulation**	Laser (λ = 786.94nm, 20 seconds/point, dosimetry = 33.5 J cm^2^, energy = 1J, 12 acupuncture points of application)^311^	76 children Laser *vs.* no laser *vs.* occlusal splint *vs.* control 12 sessions 2x/week	Laser or occlusal splint: ↓Headache ↓Bite force ↑Salivary cortisol

Notes: ↓ Decreased; ↑ Increased; ~ Not changed; *vs.* Versus; KT = Kinesio taping; VAS = Visual analogue pain scale; TENS = Transcutaneous electrical neurostimulation, ROM = Range of motion; SB = Sleep bruxism; PSG = Polysomnography; HRV = Heart variability; PNF = Proprioceptive neuromuscular facilitation; F = Frequency; Hz = Hertz; τ = Pulse width; mSec = Mili seconds; min. = Minute; EMG = Electromyography; # = Number.

### 9.3. Physiotherapeutic management protocol

**Figure 9:**
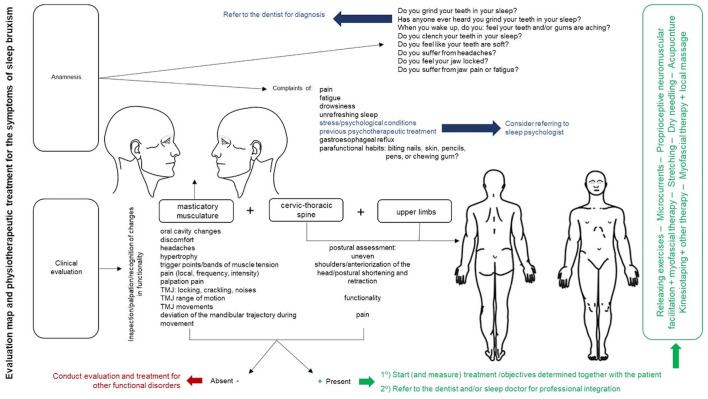
Protocol for the management of sleep bruxism by physical therapy.

The suggested protocol for the management of SB is presented in Figure 9.

Several questions remain to be answered in respect of the specific mechanisms of action of physiotherapeutic approaches to the treatment of SB, including the effect of combined therapies and multi-, trans- professional collaborative approaches.

### 9.4. Recommendations

The evidence is limited due to the small number of studies and their heterogeneity. The studies on the subject have several limitations that include a lack of standardization both in respect of therapeutic modalities and the selection of research volunteers (heterogeneity); the co-occurrence of other disorders, such as TMD. There is considerable variation in the findings and inconsistency between the studies of SB, making it impossible to perform a systematic review and meta-analysis.

However, some modalities used in PT to treat SB symptoms have been classified according to the SORT scale^2^ as detailed below in Table 13.

**Table 13:** Classification of recommendations for physiotherapeutic treatment for sleep bruxism.

Recommendation	Strength
Relaxation exercises associated with educational guidance	B
Micro currents in the muscles of the face	B
PNF associated with myofascial release (manual) and education	B
TENS (applied to the muscles of the neck and upper limb)	B
Behavioral and educational guidance^*^	B
Laser	B
Static and isometric stretching	C
Dry needling at trigger points	C
Acupuncture	C
Kinesio taping associated with other therapies	C
Myofascial release, massage	C
TENS (applied to the face)	Not recommended
Kinesiotaping (standalone therapy)	
Therapeutic exercises of muscle strengthening	
Manipulation and mobilization of vertebrae	There is no evidence to support the recommendation of this practice

Notes: PNF = Proprioceptive neuromuscular facilitation; TENS = Transcutaneous electrical neurostimulation; TENS = Low intensity current, frequency 0 to 200Hz, intended for analgesia (sensory threshold); Micro currents = Low intensity current (in the range of microamperes), low frequency, intended for pain control, healing processes and edema control, as it does not excite peripheral nerves. Both can be used continuously or alternately;

* Teach the patient to recognize and reduce the symptoms of SB by changing daytime habits. The relative safety and the non-harmful nature of the physiotherapeutic practices described, they can be recommended for inclusion in SB treatment protocols to maximize the multimodal approach, even though they are not recommended as stand-alone therapies.

Regardless of the approach chosen by PT in conjunction with dentistry, we highlight the importance of providing guidance and education about SB (and sleep hygiene) and helping patients to identify and address stressors in their routine that may contribute to their SB.

## 10. SLEEP DISORDERS RELATED TO CIRCADIAN RHYTHMICITY

Sleep disorders related to circadian rhythmicity are a distinct set of conditions primarily caused by changes in the circadian timing system due to misalignment between the endogenous expression of circadian rhythms and the external environment that affect synchronization mechanisms. In disorders of the sleep-wake cycle, the time or phase of the main sleep block is advanced or delayed in respect of the desired time, gradually becomes later each day, or is irregular each day and/or occurs in the wrong circadian phase. These 4 intrinsic disorders are known as advanced phase disorder, delayed phase disorder, free-course disorder (non-24-hour sleep-wake disorder), and irregular sleep-wake cycle disorder, respectively. In addition, there are the extrinsic disorders related to jetlag and shift work^312^ (Table 14).

**Table 14:** Intrinsic circadian rhythm disorders and their characteristics.

Disorder	Most common intrinsic causes	Main features	Characteristics of sleep/actogram^*^
Delayed phase disorder of the sleep-wake cycle	Eveningness + longer circadian period + behavior in adolescents	Extension of the intrinsic circadian period The individual has vespertine habits	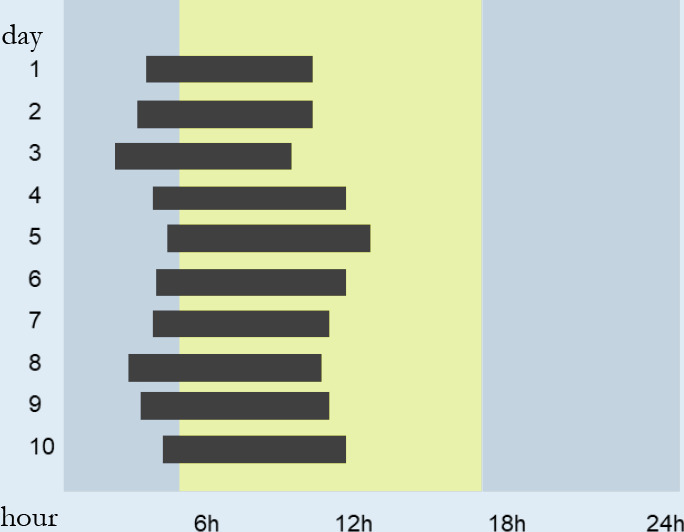
Advanced phase disorder of the sleep-wake cycle	Aging + lack of exposition to synchronizers + shorter circadian period in the elderly	The individual has morning habits	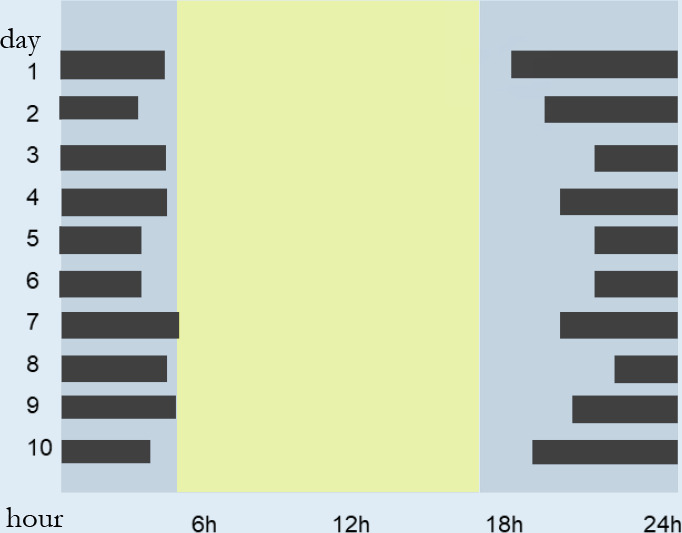
Free running or Non-24h	Frequent in the blind due to the absence of environmental light cues	The circadian system works in free running	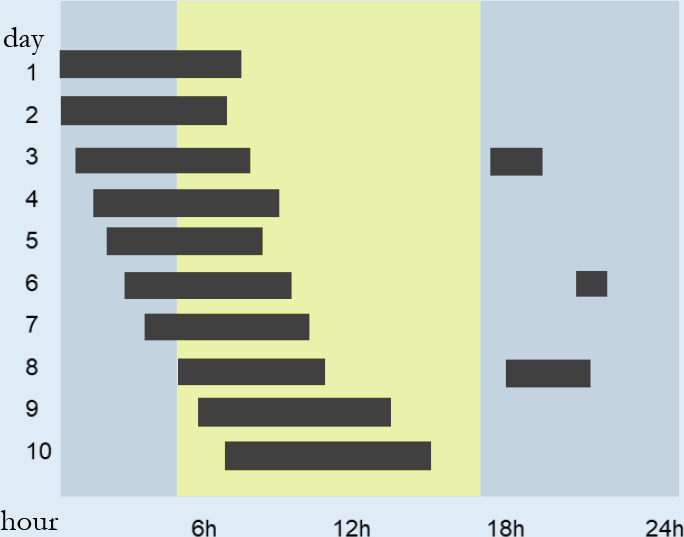
Irregular sleep-wake cycle disorder	A result of neurodegenerative conditions such as dementia	Irregular interruption of the circadian system	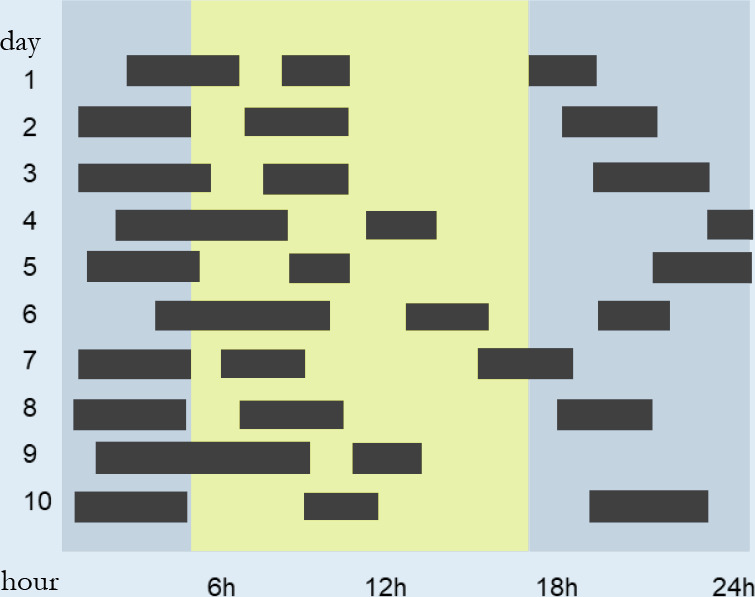

Notes:

* Each line corresponds to a day. In actograms, the vertical axis represents the days (10 days in each one of these actograms), the horizontal axis, the hours. The black bars represent the sleep phase. In blue, the dark phase (night); in yellow, the light phase (day).

It is essential to understand the concept of circadian phase and the types of stimuli to which the circadian timing system is sensitive, as the treatment of sleep disorders related to circadian rhythmicity are based on this understanding. The circadian timing system is co-responsible, along with the homeostatic mechanism of sleep regulation, for the generation of an intrinsic daily rhythm of propensity to sleep, which is linked to the natural environmental oscillations - the external synchronizers. In this way, the natural expression of the occurrence of the main sleep phase of our species is overnight, synchronized to the dark phase^313^. Light and dark are extremely cardinal signals for the correct expression of the human sleep-cycle, and act in conjunction with other temporal information, such as that arising from the routine of school, work, social relations, food cycles and physical exercise, among other synchronizers^314^. Light, both natural and artificial, interacts with the circadian timing system and generates different effects depending on the time of incidence, and its physical characteristics, such as its color, duration, and the intensity of the stimulus.

The history of chronobiology mirrors the search for understanding the interactions between light and the circadian system. Experiments first performed in animal models and later in humans showed that exposure to light was a key factor in the circadian system and that light in the morning was linked to awakening, while exposure to light in the dark phase and close to the sleep start time could delay the cycle phase and promote later sleep. These experiments showed that light was an significant synchronizer of circadian rhythms that was capable of modulating the phase of the timing system, promoting earlier or later sleep, in addition to serving as a beacon for the organism expressing its rhythmicity outside the period of 24 hours or irregularly^315^. Reveals, a window of opportunities for the performance of the PT, such as temperature and light itself, being able to research and apply the physical means under the chronobiological optics.

The property of light relates to the sleep schedule, defined by the phase or time of sleep. Its therapeutic use coincides with the very recognition of the existence of sleep-wake cycle disorders associated with circadian rhythmicity. Avoiding exposure to light, which is characterized as a stimulus, is an important tool with therapeutic potential.

In short, the etiology and pathophysiology of sleep disorders related to circadian rhythms are poorly understood. There is no consensus on the diagnostic criteria and the effectiveness of existing treatments, such as phototherapy and chronotherapy, which needs to be confirmed by more robust investigations before they are into incorporated into standard practice. The treatment of circadian rhythm disorders should be individualized^316^.

### 10.1. Advanced phase disorder of the sleep-wake rhythm

Characterized by the occurrence of the main block of sleep in a very advanced phase, usually occurring 2 or more hours before the necessary or desired time. Those affected usually complain of a very early awakening and/or have sleep-maintenance insomnia symptoms with excessive sleepiness in the late afternoon^317^. In order to delay the sleep phase, shifting the night sleep block to a later time to match conventional phases may help. The basic treatment strategy employed over the last decades has focused on the use of stimuli applied in the sensitivity window of the timing system, to delay the phase. This has been based on the application of intense light (around 4,000 lux) of 2-3 hours of duration, between 20:00 and 23:00, and ending one hour before the usual sleep time. Although this phototherapy is the main option recommended by specialists, the evidence for its effectiveness is very low (Table 14).

### 10.2. Delayed phase disorder of the sleep-wake rhythm

Characterized by the occurrence of the main block of night sleep in a very late phase, usually occurring 2 hours or more after the conventional or usual time. Those affected usually report difficulty in initiating sleep early enough to ensure an adequate duration of night sleep, and experience difficulties waking up at times compatible with the demands of work/study^317^. Like the approach used in the phase advancement disorder, treatment is focused on timing system sensitivity. In this case the photic stimulus is applied with an intensity of around 2,000-5,000 lux at dawn, starting at the time of awakening and lasting for a period of between 1-3 hours. However, the quality of the evidence of its effectiveness is very low, despite the results on sleep latency and quality^318^ (Table 14).

### 10.3. Disturbance of non-24-hour sleep-wake rhythm or free running

Characterized by the inability of individuals to synchronize the expression of the sleep-wake rhythm to the environmental synchronizers, especially to the light-dark cycle. These patients exhibit progressive phase delays in biological rhythms due to the endogenous expression of the timing system having a period which is usually longer than 24 hours. This condition is often found in blind patients without light perception, and is presented as excessive sleepiness, sleeping during the light phase and nocturnal insomnia, although a significant proportion of blind patients remain adjusted at 24 hours^319^. In recent decades, the therapeutic approach has been based on the basic theories of synchronization of biological rhythms if these rhythms can tend to run freely without synchronizing signals from the light-dark cycle. Other signals such as mealtime and social synchronizers can have a role in maintaining temporal health, and their reinforcement can be used as a non-pharmacological therapy. It is recommended that a similar approach to that used with patients with phase delay disorder is adopted, as most of these patients have an endogenous period slightly longer than 24 hours. In patients with light perception, this approach consists of the use of intense light when waking up. Although with low evidence of efficacy, the existing approach for blind adults is to promote the reinforcement of signaling through the timed use of melatonin (prescribed by physician) (Table 14). The use of melatonin is beyond the clinical scope of this consensus.

### 10.4. Irregular sleep-wake rhythm disorder

Characterized by the absence of a clear daily expression of sleep-wake behavior. Patients with neurodevelopmental, neurodegenerative, or neuropsychiatric conditions, with lesions or tumors of nervous tissue may present wakefulness and sleep dispersed in multiple blocks over 24 hours. Unlike the non-24-hour sleep-wake rhythm disorder, the irregular expression of the sleep-wake rhythm can result not only from changes in synchronizing signaling, present as a factor in the pathophysiology, but also through changes in the oscillators of the system, such as the suprachiasmatic nuclei of the hypothalamus, or in its output signal, which is fundamental for the synchronization of the other tissues of the organism^320^. Despite the many knowledge gaps about this disorder in respect of its etiology, pathophysiology and the most effective therapeutic approaches, the basic strategy of action is focused on strengthening the daily routine, with greater social exposure and a reduction of light and noise when near the sleep phase. The adoption of timed physical activity and exposure to light, preferably natural morning light of between 2,500 and 3,000 lux in intensity, is recommended, although the evidence to support the effectiveness of this approach is limited (Table 14).

### 10.5. Sleep disorders related to shift work and jetlag

Shift work and jetlag represent conditions that emerge from the exposure of the human organism to the temporal challenges imposed on modern society, since it may demand wakefulness at a time when alertness is not optimal. These challenges have the potential to disrupt the temporal organization of our organism by altering the expression of biological rhythms, such as the sleep-wake cycle and routines related to eating and physical activity. Workers on night shifts are expected to be active at time that is not biologically appropriate, resulting in inadequate, disturbed sleep (i.e., short and superficial), excessive sleepiness in the waking phase that coincides with work, often resulting in impaired performance and safety. The inversion of sleep time increases health complaints in this population^321^.

Light can be used to modulate the circadian timing system and can change the phase of the system, producing sleep advancement or delay. Light-based approaches have been used throughout history to improve the adaptation of the human organism to the challenges of shift work. The main objective of this strategy is to facilitate the adjustment of the phase of the timing system to match the period of work and to sustaining circadian alignment and avoid the rupture of the circadian timing system. High intensity, long exposure to the blue spectrum of light produces the greatest effect in terms of phase adjustment^322^. Used acutely, light also has a direct effect on the activity of the nervous system, raising alertness, with the magnitude of effect being dependent on the intensity and color of light^323^. One protocol proposes the continuous or intermittent use of light with intensities ranging between 5,000 and 1,0000 lux during work, and ending up to 2 hours before the end of the work shift, with workers avoiding exposure to natural light between the end of the night shift and the beginning of sleep^320^. However, there is limited evidence that avoiding exposure to natural light at the end of the shift is effective, and it is possible that this approach, which prevents the direct effect of acute light on alertness, can compromise safety when travelling between work and home. Long-term exposure to high-intensity artificial light is associated with an increased risk of cancer and can cause damage to the retina^321^.

Jetlag is related to the crossing time zones due to transmeridional flights, as it occurs a breakdown of internal temporal organization caused by abrupt exposure to circadian synchronizers (*zeitgebers*) at the place of arrival of the trip^324^. The number of days required for resynchronization of the timing system is proportional to the number of time zones that were crossed, and it generally takes longer after flights travelling east^319^. Emphasize that the symptoms of jetlag go beyond disturbances to the circadian timing system and sleep, generating fatigue, cognitive changes (especially when jetlag occurs chronically, as in aviation workers), mood changes and impaired gastrointestinal function^325^. Strategies to reduce the effects of jetlag aim to minimize its deleterious effects by accelerating resynchronization and thus reestablishing internal temporal organization as soon as possible^326^. Traveling to the east generates the need to advance the phase of biological rhythms, since the traveler is exposed to an advanced light-dark environmental cycle at their destination in comparison with their place of origin. Strategies to mitigate the effects of this resynchronization involve exposure to intense light (3,000 lux), continuously or intermittently, in the morning for a number of days before the trip. When arriving at the destination, the traveler must maintain exposure to natural light during the afternoon, but avoid the morning phase, since this, at the destination, would have the potential to delay the biological rhythms^327^. Symptoms associated with flights to the west are usually less intense^328^ as it is easier for humans to delay the phase of the circadian timing system than to advance it; however, flights to the west can still generate symptoms, particularly in respect of difficulties in maintaining sleep or waking early. The chronobiological approach in this case would be to increase exposure to light before arriving at the destination, preferably natural high intensity light in the late afternoon with the aim of delaying the onset of sleep. In this case, it is relevant to remain in the dark phase and avoid light in the morning^319,327^.

Other nonphotic stimuli, such as those from time-delayed physical activity, have been little explored in the literature and the results are contradictory so are not currently recommended for the treatment of symptoms associated with jetlag. There is some evidence from human studies and animal models that suggest that physical exercise may be able to advance or delay biological rhythms, such as the sleep-wake cycle^329^.

### 10.6. Physiotherapeutic objectives

- Apply light and dark at appropriate times as a therapeutic resource according to circadian rhythm disorders of the sleep-wake cycle
- Establish sleep and wake times (chronotherapy) as a strategy for circadian rhythm disorders of the sleep-wake cycle
- Recommend sleep hygiene to improve sleep efficiency and quality
- Encourage the practice of physical exercise to improve the general health of the patient, which can help to improve sleep patterns

### 10.7. Role of the physical therapist

Light and dark are synchronizers of biological rhythms, but habits and lifestyle can also impact circadian rhythms. This item will be directed to complementary guidelines to make the physiotherapeutic approach more efficient and complete^330^. The considerations in this session are important for people in general, but there is insufficient evidence for patients specifically with rhythm disorders. The timing of food consumption and its composition are essential in the synchronization of circadian rhythms since these acts as cues to a network of biological clocks throughout peripheral tissues^331^. Feeding/fasting cycles are relevant cues for the synchronization of these clocks, which are sensitive to the composition of food and the timing of its consumption and can even influence gene expression^332^. The consumption of inappropriate foods or of foods at the wrong time can result in circadian rhythm disorders. It is crucial to encourage individuals to follow consistent eating patterns to help maintain circadian rhythm and to gain the maximum benefit from treatment for synchronization. Eating and exposure to artificial light late at night can contribute to circadian desynchronization^333^. Although there are no strong evidence-based recommendations, there is a basic consensus that meals should be eaten mainly during the daytime period and eating too much around bedtime (a minimum of 2 hours before going to bed) should be avoided, as should smoking and the consumption of stimulant drinks, such as caffeine^334^.

Physical exercise can bring benefits to the patient. An active lifestyle improves overall health and prevents some sleep-related illnesses; however, exercise around bedtime should be avoided due to the alertness that it produces^321^. Activity should be of short or medium duration (30 minutes to 1 hour) and of low or medium intensity, as long-term high-impact exercises can lead to fatigue and sleep disorders^334^.

Sleep hygiene is recommended to improve the efficiency and quality of sleep^335^. For more details, see the Section 11.

Below is a summary in respect of the management of patients with circadian rhythm disorders detailing suggested steps from investigation to physiotherapeutic intervention. For example, to investigate wake and sleep times a sleep diary and actigraphy can be used. According to the results obtained, an intervention appropriate to the type of circadian rhythm disorder identified should be undertaken (Table 15).

**Table 15:** Management of patients with suspected circadian rhythm disorders.

Investigate (what)?	How to investigate?	Conduct	Physiotherapeutic intervention
Bedtime and wake up time	Sleep diary Actigraphy	Guidance on sleep/ wake up times	Light and chronotherapy suitable for the type of circadian disorder (as shown in Table 16) should be performed
Circadian preference	Questionnaire of chronotype^53^	-	
Routine and habits of life	Activity log	Guidance on routine and daily habits See Section 10.7	

### 10.8. Recommendations

PT for the treatment of circadian rhythm disorders may include modalities such as chronotherapy and phototherapy, as well as light exposure and therapeutic exercise and PT session. These modalities are classified below according to the SORT scale^2^ (Table 16).

**Table 16:** Classification of recommendations for physiotherapeutic treatment for circadian rhythm disorders.

Recommendation	Strength
*Delayed phase disorder* Light: Up to 2h exposure to natural light or ~1,000 lux in the morning. Chronotherapy: gradually advancing the sleeping time.	There is no evidence to support the recommendation of these practices
*Advanced phase disorder* Light: (1-2h from ~ 4,000 lux between 20h-23h at night). Chronotherapy: gradually delaying bedtime.	
*Non-24-hour sleep-wake disorder* No recommendation for non-pharmacological treatment.	
*Irregular sleep-wake cycle* Light: 1-2h from 2,500 to 5,000 lux, between 9:00 and 11:00. Chronotherapy: insert sleep and wake-up routine and low-intensity exercise.	

Note: Based on American Academy of Sleep Medicine Clinical Practice Guidelines^336^.

There is a need for further research in this field since the literature presents conflicting data and lacks studies with sufficient methodological rigor. Because some treatment is based on low-cost therapeutic approaches, such as the use of light, it would be highly cost-effective. Deepening knowledge in this field would greatly benefit patients affected by these disorders and open an avenue for future treatments.

## 11. INSOMNIA

Insomnia is a sleep disorder that should be regularly investigated by physical therapists, as it can affect an individual’s overall physical condition. Health problems presented by the patient, whether this be pain, limitation of movement, fatigue or other conditions, can negatively influence sleep. When treating patients after an injury, PTs should routinely ask whether there have been any changes in sleep pattern following the injury in order to guide the direction of the intervention and provide the most effective treatment^87,337,338^.

### 11.1. Insomnia and physical activity

The Brazilian Ministry of Health^339^ recently published the *Physical Activity Guide for the Brazilian Population* which states *“The earlier physical activity is encouraged and becomes a habit in your life, the greater the benefits for your health*”. In this guide, improved sleep is highlighted as one of the benefits of physical exercise. Exercise is proposed as a non-pharmacological alternative for the treatment of insomnia because it is characterized as a safe and accessible strategy that is capable of improving sleep^340^.

### 11.2. Insomnia and pain

A changed sleep pattern is one of the most frequent complaints of patients with pain conditions. There is an intrinsic relationship between pain and sleep, with pain interfering with the quantity and quality of sleep, and impaired sleep triggering or aggravating pain^341^. The presence of sleep disorders and the number of health complaints predict the onset, persistence and worsening of pain^13,342-344^.

### 11.3. Insomnia and other comorbidities

Insomnia can result from inappropriate habits, or from a disease, being either a symptom or a disorder. PTs need to identify and evaluate insomnia in patients so that it can be monitored to avoid aggravating fatigue, reducing quality of life or having an impact on the rehabilitation process^345^. Poor sleep quality is associated with the accumulation of beta-amyloid protein and the consequent development of neurological diseases, such as Alzheimer’s disease^346^. Improving sleep quality can promote positive and immediate effects on the quality of life of the patients^347^. Optimizing sleep, as early as possible in the life of any person, is a way to prevent or delay the development of various diseases and effectively improve the rehabilitation process when illness does occur.

### 11.4. Physiotherapeutic objectives

- Identify which signs and symptoms presented by the patient are related to insomnia;
- Make the patient aware that insomnia can negatively affect their physical and mental condition, and their health in general;
- Make the patient aware that their condition (e.g., physical, pain, and fatigue) can interfere with the quality and quantity of sleep;
- Provide the patient with guidance about habits that can be adopted to improve sleep;
- Present physiotherapeutic treatments to the patient that can improve their sleep.
- Assist in the treatment of insomnia by interacting with the medical team.

### 11.5. Role of the physical therapist

Over the years, several non-pharmacological treatments for insomnia have shown promising results. One of these is therapeutic physical exercise (Table 17). Other physical therapy modalities are presented on Table 18. If the patient does not have a formal diagnosis and treatment of insomnia, refer the patient to the sleep physician to guide treatment.

**Table 17:** Types of therapeutic physical exercise to be considered in the treatment of insomnia and their parameters.

Modality	Aerobic exercise Resistance and muscle strength exercises Stretching, Yoga, Tai Chi Chuan Increase the daily steps/movement Regular physical activity: walking, dancing, cycling, etc. Consider type of exercise and environment (indoor/outdoor)
**Intensity**	Moderate to intense
**Duration**	Minimum of 50 minutes (3 times a week) or at least 150 minutes a week
**Time of the day**	It should be defined individually, preferably considering the circadian preference Physical exercise, especially high intensity, should be avoided very close bedtime (as exercise can produce a state of alertness)
**Length**	Minimum of 2 months for subjective sleep improvement; from 4 months there is systemic improvement, leading to a reduction in hypervigilance Make therapeutic exercise a habit to maintain its effects and avoid the recurrence of symptoms

Note:

* These recommendations cannot be generalized, as chronotypes, circadian preferences and behaviors need to be taken into account.

**Table 18:** Physical therapy interventions for patients with insomnia.

Modality	Parameters/protocol/reference	Outcomes
**Exercise**	17 sedentary adults with insomnia^357^ 16wk of PA, 4x/without PA **Gr1:** Aerobic PA **Gr2:** Aerobic PA + SH	**Gr2:** ↓PSQI ↓ESS
	48 patients with insomnia (38 women)^355^ Acute intervention (single session) **Gr1:** Aerobic PA, high intensity **Gr2:** Aerobic PA, moderate intensity **Gr3:** Resistance PA, moderate intensity **Gr4:** Control	**Gr2:** **PSG:** ↓SL ↓WASO ↑TST ↑SE **SD:** ↓SL ↑TST
	19 sedentary adults with insomnia^356^ 6 months, 3x/without **Gr1:** Aerobic PA, moderate intensity (morning) **Gr2:** Aerobic PA, moderate intensity (evening)	**Gr1 and Gr2:** **PSG:** ↓SL ↓WASO ↑SE **SD:** ↓SL, ↑ sleep quality ↑Feeling of rest upon waking
	173 sedentary, overweight or postmenopausal women^379^ **Gr1:** Aerobic PA: 12 months, 5x/wk,45min/session **Gr2:** Stretching exercises: 12 months, 60min. of stretching 1x/without supervision and 15-30min, 3x/without supervision at home	**Gr1 and Gr2:** ↓Drugs used to help initiate sleep
	28 sedentary adults with insomnia^11^ 4 months, 3x/wk, between 17 and 18h **Gr1**: Resistance PA (first 2 months with 50% of 1MR and last 2 months with 60% of 1MR) **Gr2:** Stretching exercises **Gr3:** Control	**Gr1 and Gr2:** **Act.:** ↓SL ↑SE ↓WASO ↓ PSQI ↓ ISI
**Acupuncture**	28 participants with breast cancer, in chemotherapy or after chemotherapy, with insomnia^380^ 6wk, 2 x/without **Gr1:** Electroacupuncture + auricular acupressure **Gr2:** Control	**Gr1:** ↓ISI ↓PSQI **SD:** ↑TST
	20 participants with cancer and insomnia^381^ 4wk, 10 sessions, 2 to 3x/wk, 30min./session **Gr1:** Electroacupuncture **Gr2:** Simulated electroacupuncture **Gr3:** Usual care	**Gr1:** ↓ISI PSQI **SD:** ↓SL ↑TST ↑SE **Act:** ↓SL
**Acupressure**	114 participants with cancer in chemotherapy, with insomnia^382^ 4wk Auto acupressure at home, 30 to 60min. before bedtime **Gr1:** True acupressure **Gr2:** Simulated acupressure **Gr3:** Control	**Gr1 e Gr2:** ↓IGI ↓Anxiety ↓Depression
	200 participants with insomnia. 4 without from 10 to 15min. every night^383^ **Gr1:** Auto acupressure **Gr2:** SH	**Gr1:** ↓ISI ↓Anxiety ↓Depression
**Massage**	44 postmenopausal women with insomnia^384^ 16wk, 32 sessions, 2x/week **Gr1:** Therapeutic massage **Gr2:** Passive exercise **Gr3:** Control	**Gr1:** ↓ISI ↑Quality of life ↓Symptoms of depression
**Phototherapy**	56 participants with insomnia, post mild to moderate stroke^385^ 2wk, 30min./day **Gr1:** Phototherapy (10,000 lux) in the morning (between 7:00 and 8:00) **Gr2:** Placebo therapy (50 lux)	**Gr1:** ↓IGI **Act:** ↓SL ↑SE ↓ESS ↑Quality of life ↓Symptoms of depression
	30 participants (20 women) with insomnia^369^ 60 days in the morning **Gr1:** 10,000lux/20min. **Gr2:** 10,000lux/45min.	**Gr2:** Best responses with 3 and 6 months follow-up **SD e Act:** ↓SL ↑TST
	140 patients (49 women) with Parkinson’s disease^386^ Light therapy for 34 days, 60min./day between 7 and 8 p.m.	↑TST ↓Number of awakenings ↓WASO ↓ISI (in 5 years)
**Yoga**	44 women with postmenopausal insomnia^352^ 4 months, 1x/without, 1h session **Gr1:** Yoga **Gr2:** Passive stretching	**Gr1:** ↓ISI ↓Symptoms of menopause ↑Quality of life
	44 people with insomnia^375^ 8wk, 60min./day **Gr1.:** Yoga **Gr2:** SH	**Gr1:** **DS:** ↓SL ↑TST ↑SE ↓ISI
	41 participants, with insomnia^387^ 4wk Gr1: Yoga Gr2: Cognitive behavioral therapy	**Gr1 and Gr2:** **PSG:** ↑TST ↑ N1% **SD:** ↑TST ↑SE ↓ WASO **Yoga:** ↑%N2 ↑%N3 ↓Salivary cortisol
**Tai Chi Chuan**	123 participants, with insomnia^354^ 4 months, 1x/wk, 120min., in group **Gr1:** TCC **Gr2:** Tai Chi Chuan **Gr3:** SH	**Gr1:** remission of insomnia **PSG:** ↓SL ↑SE ↓WASO ↑Sleep quality, fatigue, and depressive symptoms **Gr2:** ↑The overall quality of sleep and fatigue compared to the SH group
	320 participants (80% women), with insomnia^388^ 12wk, 3x/wk, 60min./session (group of 6 or 15 participants) **Gr1:** Conventional exercise training (brisk walking and muscle-strengthening exercises) **Gr2:** Tai Chi Chuan	**Gr1 and Gr2:** **Act.** (7 days) ↑SE ↓WASO

Notes: PA = Physical activity; PSG = Polysomnography; SD = Sleep diary; SL = Sleep onset latency; WASO = Wake after sleep onset; TST = Total sleep time; SE = Sleep efficiency; PSQI = Pittsburgh sleep quality index; ISI = Insomnia severity index; SH = Sleep hygiene; Gr. = Group; ESS = Epworth sleepiness scale; Act = Actigraphy; MS = Muscle strength; wk = Week; MR = Maximum resistance.

#### 11.5.1. Therapeutic physical exercise

The *European Guideline for the Diagnosis and Treatment of Insomnia*^348^ recommends physical exercise to be performed as an adjuvant treatment of patients with insomnia. Physical exercise can be an important non-pharmacological intervention to improve insomnia. In general, the reliability of the studies has been found to be reduced by methodological limitations, limiting their generability^349-351^, although some studies have indicated promising results^11,352-357^.

Resistance exercise has been demonstrated to improve neuroplasticity^358,359^, thereby improving synaptic functioning of brain areas related to anxiety^360^. A reduction in symptoms of anxiety and a state of hypervigilance tends to improve sleep. Some systematic reviews with meta-analysis have extensively explored this subject. A program of moderate intensity physical exercise in middle-aged women improved the quality of sleep but did not alter the severity of insomnia^351^. Physical exercise was shown to improve sleep quality, without triggering important adverse effects in patients with insomnia^349^. The practice of physical exercise improved subjective sleep quality in people with insomnia (symptom and disorder), but the objective improvement was observed only in individuals with insomnia symptoms^350^. There is evidence of the benefits of regular practice (duration of at least 2 months) of different types of physical exercises (physical and body-mind) in the quality of sleep and insomnia^361^. The most recent systematic review on the effects of exercise identified improvements in subjective sleep parameters, as well as decreased severity of insomnia, with a moderate power of effect^362^. However, the authors did not observe a statistically significant difference in objective sleep variables^362^.

It is not yet possible to define the most effective protocol for the treatment of insomnia using exercise. Characteristics related to the different types of exercises should be taken into account^363^, without disregarding the variables related to the individual, such as self-efficacy, the social aspect and the pleasure associated with some types of exercise.

#### 11.5.2. Acupuncture and acupressure

Acupuncture has been a regulated practice in PT in Brazil^364^. This ancient technique has been indicated to be effective in the treatment of chronic insomnia in several systematic reviews^365,366^, but without consensus on the acupuncture points, number or duration of the sessions.

#### 11.5.3. Massage

The effects of massage can be transmitted to the central nervous system through receptors of touch, pressure, heat and vibration, generating a feeling of relaxation, tranquility, calm and sleep. Massage has the advantage of being simple and without side effects, and, in general, is a low-cost intervention with significant benefits for patients with insomnia^367,368^.

#### 11.5.4. Phototherapy

This is a promising technique and can be used concurrently with other treatments^348^. It is still necessary the research in specific populations to better understand their mechanisms. Light therapy consists of the emission of radiation with a blue wavelength. For the treatment of insomnia, 10,000 lux is used, usually positioned at eye level, at a distance of approximately 75cm, with exposure time ranging between 30 minutes and 2 hours^369,370^.

#### 11.5.5. Relaxation

There are several muscle relaxation techniques, such as progressive muscle relaxation^371,372^, autogenic training^373^, Yoga^374,375^, mindfulness^376,377^ and imagery^378^. It is recommended to be performed once during the day and repeated immediately before bed, preferably lying down. Practice is critical, as so is regularity, no matter which relaxation method is used, as therapeutic benefits are largely to emerge^378^.

### 11.6. Recommendations

PT for insomnia includes a range of modalities (Table 19) classified according to the SORT scale^2^.

**Table 19:** Classification of physiotherapeutic strategies for the treatment of insomnia based on their strength of recommendation taxonomy scale classification.

Recommendations	Strength
Aerobic exercise as an adjunct treatment^*^	B
Resistance exercise as an adjunct treatment^*^	B
Acupuncture	C
Massage	C
Muscle relaxation	C
Yoga	C
Tai Chi Chuan	C
Sleep hygiene	C
Phototherapy	There is no evidence to support the recommendation of these practices
Ergonomics of sleep	

Note:

* However, there is no consensus on the type of exercise in respect of duration, frequency, intensity, time to be performed, environment (whether outdoors or indoors), with or without supervision, or individually or in a group.

#### 11.6.1. Ergonomics of sleep

It is not uncommon for people to wake up with some kind of pain or discomfort after a bad night’s sleep. In many cases, these problems can easily be avoided by providing suitable guidance about sleep, particularly in respect of the ergonomics that surround it, namely the positions in which we choose to sleep and the accessories we use (pillows, mattress), that can be a significant factor in producing unwanted pains. It is difficult to determine the ideal mattress or pillow, or the best posture to adopt for sleep as there is such a multitude of variables that need to be carefully evaluated before the best “ergonomic protocol” can be identified. Considering about the anatomy and biomechanics of the spine and sleeping, positioning can be a starting point, for those with positional sleep apnea (more details in the Sections 5.3.3 and 6.8), and especially for people who already suffer from some pain condition.

Lateral decubitus: this is usually the most recommended posture to avoid a number of problems, especially in the spine. When well adopted, it allows a better distribution of loads along the body, with better alignment of the column. In this position, the ideal is for the pillow to fill the entire distance between the mattress and the ear, so that the head maintains a good alignment with the rest of the spine. A pillow should be used between the knees to level the distance between the knees and hips, thereby avoiding rotation of the trunk. If preferred, a pillow can be hugged to allow greater relaxation of the posterior muscles and better support of the upper limbs.

Another factor that has been studied in respect of sleeping position is its influence on the glymphatic system, as the action of gravity can interfere with cerebral blood flow and affect the elimination of waste products from the brain. In humans, some studies have suggested that there is an association between sleep in dorsal decubitus for more than 2 hours per night and the development of neurodegenerative diseases^389^. In addition, in animals, the efficiency of the glymphatic system has been shown to be higher in lateral decubitus^390^. Epidemiological studies pointed that most people sleep in this position, which offers significant protection against cervical, scapular and arm pain, and generates better sleep quality^391^.

Dorsal decubitus: this may provide an acceptable alternative posture, but some care needs to be taken; a pillow (small) should be used under the head, and another should be placed under the knees to allow a small degree of flexion, and, less overload on the lumbar spine.

Ventral decubitus: this posture should be avoided by most people because it places the cervical region in maximum rotation and the lumbar spine in hyperlordosis. One way to minimize this condition is to place a pillow under the pelvic region, although the cervical spine will still be in an unfavorable position. Sleeping in a prone position is significantly associated with an increased prevalence of all categories of pain and reports of lower quality of sleep^391^.

#### 11.6.2. Sleep hygiene

Sleep hygiene (SH) is a set of instructions on sleep habits and behaviors, which aims to improve the quality and quantity of sleep and is another strategy that requires further investigation in respect of its effectiveness in the treatment of insomnia, including through sleep restriction therapy. Although widely advocated, it has difficulties in clinical practice in respect of how to approach the patient and encourage them to practice it routinely at home. Educational programs should be used to promote changes in the sleep habits of patients, and when necessary, they should be referred to a sleep psychologist for the gold standard therapy - cognitive behavioral therapy for insomnia (CBT-i)^363,392-394^. In Brazil, CBT-i is a scope of practice of psychologists specialized in this area. Some SH recommendations are described below in Table 20^395-398^.

**Table 20:** Sleep hygiene recommendations.

Routine	Maintain regular schedules, including at weekends. Exposure to morning sunlight helps wake up.
**Use of bed**	Use the bed for sex and sleep. Avoid working and eating in this environment.
**Difficulty returning to sleep**	When awakening in the middle of the night and having difficulty returning to sleep within 30 minutes, leave the room and perform activities of low brain stimulation, returning to the room only when sleepy.
**Pre-sleep routine**	Create a pre-sleep routine to condition the brain for bedtime. Decrease stressful activities, reduce contact with electronic devices that emit lights and do something pleasurable and relaxing (hot bath, meditation, mindfulness, stretching or reading).
**Physical activity**	Practice physical activity and avoid sedentary behavior. Regular exercise improves sleep quality, decreases excessive sleepiness and makes people more energetic throughout the day. Some guidelines suggest avoiding the practice of high intensity physical exercises, at least 2-3 hours before sleep, as it could stimulate brain pathways^399^. However, evidence suggests that this may only be relevant to some people, and individual circadian preferences should be considered before applying any specific guidelines.
**Stimulants**	Avoid the consumption of caffeinated beverages, stimulants, cigarettes and alcoholic beverages at least 4 hours before bedtime. Caffeine can cause difficulty falling asleep because it competes with adenosine diphosphate receptors, preventing it from binding to neurons, hyperpolarizing them and inducing sleep^400^. The consumption of alcoholic beverages, in general, generates the false impression of helping sleep. Consumption can help to induce sleep, but it favors a more superficial and fragmented sleep. Nicotine acts as a stimulant, keeping the central nervous system in a state of prolonged alertness. In addition, the release of melatonin, an essential hormone for sleep, is deregulated by the presence of nicotine because it can promote increased secretion of adrenaline, which is a stimulant.
**Naps**	Avoid napping during the day. In general, people who sleep well will not feel the need for daytime sleep. But if there is a need, take a nap at a single time of the day, preferably after lunch with a maximum duration of 30 minutes.
**Light**	Do not use appliances that emit light approximately 1 hour before the time you wish to sleep. Remember that melatonin is secreted in the absence of light.
**Environment**	Take care of the details that make up the sleeping environment, making it comfortable, relaxing, and safe space. Control the entry of light (curtains), noise (anti-noise windows) and temperature (air conditioning, fan, and covers). If necessary, use an eye mask and earplugs. Extreme temperatures can interfere with sleep quality.
**Eating**	Avoid eating large quantities of food, particularly high-calorie foods, around the time you want to sleep. The ideal is to eat at least 2 to 3 hours before going to bed. Physiologically, the rhythm of the digestive system will be reduced at night. Beware of excessive fluid intake around bedtime to avoid nighttime awakenings due to increased diuresis.

These non-pharmacological strategies suggested for the treatment of insomnia can produce satisfactory results for patients, especially for those with inadequate and harmful sleep habits.

## 12. WILLIS-EKBOM DISEASE AND PERIODIC LIMB MOVEMENT DISORDER

To date, the treatment for Willis-Ekbom disease (WED)^20,401^, which is known as restless legs syndrome (RLS), and for periodic limb movements during sleep^402^ (PLMS) is pharmacological and non-pharmacological^402^, with physiotherapeutic interventions used as an adjunctive therapy. Despite being 2 different entities, both phenomena often occur simultaneously, and the absence of WED is a criterion for the medical diagnosis of periodic limb movement disorders (PLMD). In this consensus, we are using the WED nomenclature for the condition rather than for RLS, because the disease can affect the arms as well as the legs.

The symptoms of WED and PLMD (Table 21) can be treated by non-pharmacological agents, although there is low-quality scientific evidence of its effectiveness^402^.

**Table 21:** Symptoms of Willis-Ekbom disease and periodic limb movement disorder.

Willis-Ekbom disease^401^		Periodic limb movement disorder^20^
Urgency to move legs, arms, or less commonly other parts of the body		Repetitive spasms or kicks (usually every 20 to 40 seconds) of the lower or upper limbs during sleep
Rest or inactivity precipitates the sensation		
A feeling of anguish, anxiety, despair, sadness, and distress		Unrefreshing sleep
Colic	A feeling of heaviness	Sleep fragmentation
Irritation	A prickling sensation	-
Itching	Burning, heat	-
Numbness	“Cold in the bones”	-
Pain	Desire to move	-
Tickling	Muscle contraction	-
Tingling	Restlessness	-
Tiredness	Strangulation, contraction	-

### 12.1. Physiotherapeutic objectives

- Relieve the symptoms;
- Decrease the severity of symptoms;
- Educate patients about the disease and its management, and the importance of therapeutic exercise and daily physical activity;
- Promote improved sleep hygiene to avoid aggravating symptoms;
- Improve the quality of sleep;
- Improve the quality of life.

### 12.2. Role of the physical therapist

First, the interaction with the medical team for receiving supportive information about the proposed treatment is crucial. Second, several studies have shown significant improvements in the symptoms of WED and PLMD following the use of non-pharmacological therapies for their treatment^403-408^. Since WED improves with movement, the hypothesis that exercise improves the disease has been tested, with promising findings in different populations (Table 22). Until now, we do not know the precise mechanisms through which exercise improves WED symptoms, and there is not enough evidence in the literature to support the use of non-pharmacological treatments as standalone treatment.

**Table 22:** Physiotherapeutic treatment of Willis-Ekbom disease and periodic limb movement disorder.^*^

Physiotherapeutic modality		Parameters	Outcomes
**Acupuncture**	Acupuncture^409^	3x/week, 30min.	**↓** WED severity (actigraphy)
	Acupuncture + gabapentin *vs*. gabapentin^410^	6 weeks, 3x/week 60min.	Acupuncture + gabapentin: **↓** WED severity ↑ Sleep quality Gabapentin: **↓** WED severity
	Acupressure (CKD population)^411^	4 weeks, 3x/week, 36min. During the hemodialysis sessions	**↓** WED severity ~ Quality of sleep
**Therapeutic exercises**	Aerobic (CKD population)^412^	6 months, 3 x/week, 45min. 60% - 65% HR_MAX -_ ergometric stationary bike	**↓** WED severity ↑ Quality of sleep **↓** Symptoms of depression
	Aerobic (CKD population) *vs.* dopaminergic agonist^413^	6 months control *vs.* dopaminergic agonist *vs.* exercise during dialysis 60%-65% HR_MAX -_ reclined ergometric stationary bike	Both groups: **↓** WED severity ↑ Quality of sleep ↑ Quality of life ↑ Cardiovascular performance
	Stretching (CKD population)^414^	8 weeks (24 sessions), 3x/week every 30min. at the end of dialysis	**↓** WED severity
	Aerobic (CKD population)^415^	16 weeks, 3x/week, 30min. sessions performed between hour 2 and 3 of dialysis using an ergometric stationary bike	**↓** WED severity
	Aerobic (CKD population)^416^	16 weeks, 3x/week, 45min. hour 2 and 3 of dialysis, reclined ergometric stationary bike 65% to 70% resistance (watts) readjusted every 2 weeks 45 to 50rpm	↑ Quality of life
	Aerobic (spinal cord injury population)^417^	45 days, 3x/week, 30min. LV1 70-80rpm ergometric stationary bike	**↓** PLMi
	Aerobic *vs.* dopaminergic agonist (spinal cord injury population)^418^	45 days dopaminergic agonist (200mg) *vs*. exercise 3x/week, 30min. LV1 stationary bike 70-80rpm	**↓** PLMi in both protocols
	Aerobic + low resistance^419^	12 weeks, 3 x/week 40min. aerobic + 1 sets of 8 to 12 repetitions in the first 2 weeks, and after that, 2 sets of 12 repetitions Free times to attend	**↓** WED severity
	Acute *vs.* chronic exercise^420^	Acute: 3 minutes at 50 watts, with progression of 25 watts every 2 minutes, until exhaustion Chronic: 12 weeks (72 sessions), 3x/week 50 minutes at LV1, ergometric bike	Acute: ↑TST ↑SE, ↑ REM, ↓WASO, ↓PLMi Chronic: ↑TST, ↑SE, ↑ SL, ↓ REM SL, ↓PLMi
	Acute aerobic exercise^421^	1 session progressive charge 2 minutes starting at 4 km with progression of 1km/min. until exhaustion, treadmill	↑ Stage N1 ↓ PLMi
**Phototherapy**	Near infrared light^422^	4 weeks, 3x/week, 30 minutes Pulse F: 292Hz, λ=890nm, 50% cycle transcutaneous, administered via probe/diode transducer	**↓** WED severity
	Infrared light^423^	λ=850nm dosimetry=8J/cm^2^ transcutaneous, administered by probe/diode transducer	**↓** WED severity ~ VAS ~ muscle ultrasound Improvement of skin sensitivity (esthesiometry)
**Manual therapies**	Massage + heating^424^	5 days, warm massage from feet to knees	**↓** WED severity
	Massage with lavender oil^425^	3 weeks, 2x/week, 10 minutes Lavender oil massage in lower limbs, 1 hour after dialysis start	**↓** WED severity
	Massage *vs.* electric vibration^426^	1 month, 3x/week, final 10 minutes of dialysis Massage of lower limbs Electric foot massage at low voltage	**↓** WED severity ↑ Quality of sleep
**Compression therapies**	Pneumatic compression in lower limbs^427^	4 weeks, 1hr/ day, 1hr before the onset of symptoms 40cmH_2_O each compression	**↓** WED severity
**Yoga**	Iyengar Yoga^428^	8 weeks, 2 x/week, 90min + 30min, online lessons at home Iyengar Yoga	**↓** WED severity Improvement of anxiety **↓** Blood pressure

Notes:

* The modalities presented without comparisons of groups were interventions compared to control groups; ↑: Increased; ↓: Decreased; ~: No statistically significant changes; CKD = Chronic kidney disease, dialysis population; min. = Minutes; HRmax.= Maximum heart rate; TST = Total sleep time (polysomnography examination; PSG); SE = Sleep efficiency (PSG); SL = Sleep latency (PSG); REM SL = REM sleep stage latency (PSG); REM: REM sleep stage (PSG) = WASO: wake after sleep onset (PSG); PLMi = Periodic leg movement index (PSG); F = Frequency; λ = Wavelength; J = Joules; VAS = Visual analogue scale for pain intensity; rpm = Revolutions per minute; LV1 = First minute ventilatory threshold.

The most recent literature review on the subject demonstrated that therapeutic exercise, pneumatic compression, infrared light therapy and acupuncture were more effective in reducing the severity of WED when compared to control conditions^403^. Another review showed improvements in severity in investigations involving all the mentioned modalities, including phototherapy^404^. In specific conditions such as chronic kidney disease, aerobic exercises associated with resistance exercise reduced the severity of WED^405^ symptoms; but the studies comprised only small samples and the conclusions are still uncertain. A later meta-analysis in the same population confirmed that lower limb stretching exercises performed during dialysis were effective in reducing the severity of the symptoms of the disease^406^. A group of researchers investigated the therapies most commonly used by patients to improve and alleviate the symptoms of WED and found that deep massage was used as a therapy in approximately 77% of cases, although the relief was momentary^407^. According to another systematic review, Yoga and pneumatic compression improved symptoms associated with WED^404^. A 2021 meta-analysis described that a combination of pharmacological treatment with acupuncture produced better clinical outcomes for PLMD, when compared to acupuncture alone^408^. For WED and PLMD, PTs should educate patients about the condition and encourage them to take regular physical exercise and practice good sleep hygiene (as poor sleep can worsen symptoms).

The type of physiotherapy applied depends on the physical condition of the patient, (e.g., treadmill or exercise bike or upper limb cycle ergometer). There is no consensus in the literature regarding the duration, frequency and intensity of therapeutic exercise (Table 22).

### 12.3. Physiotherapeutic management protocol

**Figure 10:**
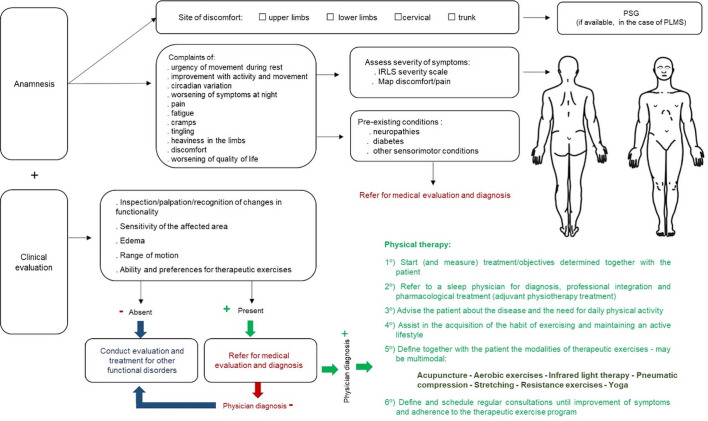
Suggested physiotherapeutic protocol for the treatment of WED and PLMD.

The sleep PT should proceed according to the physiotherapeutic evaluation, as discussed earlier in Section 3. Caution should be taken as WED can commonly be confused with other neuropathies (Figure 10). The integrative work with a physician is imperative in the treatment. The measurement of the results of the physiotherapeutic treatment using the International Restless Legs Syndrome Study Group Rating Scale^55^ is of paramount importance (more details in the Section 3).

### 12.4. Recommendations

To date, there is no scientific evidence to support non-pharmacological treatment in WED and PLMD symptoms as isolated therapy. Clinical practice suggests that the frequency of the activity is associated with the perception of the symptoms. The minimum frequency to be adopted is 3x/week, ideally the modality should be performed daily. Therapeutic exercise, preferably aerobic and resistance, except in the case of patients with CKD and on hemodialysis, are the most effective modality according to the SORT scale^2^ (Table 23), although there is still a low quality of evidence for the general population. Multimodal approaches to therapies have yet to be investigated.

**Table 23:** Classification of recommendations for physiotherapeutic treatment for Willis-Ekbom disease and periodic limb movement disorder.

Recommendation	Strength
Aerobic + resistance exercises	B
Acupuncture	C
Aerobic exercise	C
Resistance exercise	C
Infrared light/near infrared light	C
Manual therapies (massage)	C
Iyengar Yoga	C
Dry needling	There is no evidence to support the recommendation of these practices
Pneumatic compression	
Cryotherapy	
TENS	

## 13. FINAL CONSIDERATIONS

Sleep has an essential function for maintaining life. There exists an increasing incidence of sleep disturbances with a negative impact on the sleep health, quality of life and, in some cases, in life expectancy.

The main outcome of the sleep PT’s assessment is the improvement in sleep functionality and quality. The sleep PT has a significant role in sleep health team. The sleep PTs training is due to extensive knowledge related to the physiology and pathophysiology of sleep, and the intrinsic body system’s related to sleep. The sleep PT can guide the patient through the sleep hygiene and good practices related to sleep health, with the prescription and guidance of therapeutic exercises, relaxation and the use of many PTs modalities/resources/techniques.

This consensus is the synthesis of current knowledge and state of the art of sleep PT and will help in the opening of new venues for investigation within PTs scope of practice for the growth of the profession. It aims to assist the PTs in their training and development, demonstrating the best practices of evaluation and conducting interventions to restore the best functionality of the patient.

## ABREVIATION LIST

AASM: American Academy of Sleep Medicine
ABS: Brazilian Sleep Association
Act: Actigraphy
AHI: Apnea hypopnea index
ASSOBRAFIR: Brazilian Association of Cardiorespiratory Physiotherapy and Physiotherapy in Intensive Care
ASV: Adaptive Servo-Ventilation
APAP: Automatic PAP
BMI: Body mass index
CBT-i: Cognitive behavioral therapy for insomnia
CHF: Congestive heart failure
CKD: Chronic kidney disease
CO_2_: Carbon dioxide
COFFITO: Brazilian Federal Council of Physical Therapy
COPD: Chronic obstructive pulmonary disease
CPAP: Continuous PAP
CSA: Central sleep apnea
CSB: Cheyne-Stokes breathing
EMG: Electromyography
ESS: Epworth sleepiness scale
FEV_1_: Forced expiratory volume in one second
FVC: Forced vital capacity
Gr.: Group
HRV: Heart rate variability
ICD: International Statistical Classification of Diseases and Related Health Problems
ICF: International Classification of Functioning and Health
ICSD-3: International Classification of Sleep Disorders, 3^rd^ Edition
ISI: Insomnia severity index
KT: Kinesiotaping
MR: Maximum resistance
MS: Muscle strength
NIV: Non-invasive ventilation
NMDs: Neuromuscular disease
NREM: Non-REM
O_2_: Oxygen
ODI: Oxyhemoglobin desaturation index
OHS: Obesity hypoventilation syndrome
OSA: Obstructive sleep apnea
PA: Physical activity
PaCO_2_: Partial pressure of carbon dioxide on arterial blood
PAP: Positive airway pressure
PCO_2_: Partial pressure carbon dioxide
PetCO_2_: Partial end-tidal carbon dioxide
PLMD: Periodic limb movement disorder
PLMi: Periodic limb movement index
PLMS: Periodic limb movements during sleep
PNF: Proprioceptive neuromuscular facilitation
PS: Primary snoring
PSG: Polysomnography
PSQI: Pittsburgh Sleep Quality Index
PT: Physiotherapy, physical therapy, physiotherapist
PtcCO_2_: Transcutaneous carbon dioxide pressure
PTs: Physiotherapists
REI: Respiratory event index
REM: Rapid eye movement sleep
RERA: Respiratory effort related arousal
RLS: Restless legs syndrome
ROM: Range of motion
SB: Sleep bruxism
SDB: Sleep-disordered breathing
SD: Sleep diary
SE: Sleep efficiency
SH: Sleep Hygiene
SL: Sleep onset latency
SORT: Strength of Recommendations Taxonomy
SpO_2_: Oxygen saturation
TE-CSA: Treatment emergent central sleep apnea
TENS: Transcutaneous neuromuscular electrical stimulation
TMD: Temporomandibular joint disease
TMJ: Temporomandibular joint
TST: Total sleep time
UA: Upper airway
VAS: Visual analogue scale of pain
WASO: Wake after sleep onset
WED: Willis-Ekbom disease
WHO: World Health Organization
Wk: Week
